# Enhanced polygenic risk score incorporating gene–environment interaction suggests the association of major depressive disorder with cardiac and lung function

**DOI:** 10.1093/bib/bbae070

**Published:** 2024-03-02

**Authors:** Chuyu Pan, Bolun Cheng, Xiaoyue Qin, Shiqiang Cheng, Li Liu, Xuena Yang, Peilin Meng, Na Zhang, Dan He, Qingqing Cai, Wenming Wei, Jingni Hui, Yan Wen, Yumeng Jia, Huan Liu, Feng Zhang

**Affiliations:** Key Laboratory of Trace Elements and Endemic Diseases of National Health and Family Planning Commission, Key Laboratory of Environment and Genes Related to Diseases of Ministry of Education of China, Key Laboratory for Disease Prevention and Control and Health Promotion of Shaanxi Province, School of Public Health, Health Science Center, Xi'an Jiaotong University, Xi'an, P. R. China; Key Laboratory of Trace Elements and Endemic Diseases of National Health and Family Planning Commission, Key Laboratory of Environment and Genes Related to Diseases of Ministry of Education of China, Key Laboratory for Disease Prevention and Control and Health Promotion of Shaanxi Province, School of Public Health, Health Science Center, Xi'an Jiaotong University, Xi'an, P. R. China; Key Laboratory of Trace Elements and Endemic Diseases of National Health and Family Planning Commission, Key Laboratory of Environment and Genes Related to Diseases of Ministry of Education of China, Key Laboratory for Disease Prevention and Control and Health Promotion of Shaanxi Province, School of Public Health, Health Science Center, Xi'an Jiaotong University, Xi'an, P. R. China; Key Laboratory of Trace Elements and Endemic Diseases of National Health and Family Planning Commission, Key Laboratory of Environment and Genes Related to Diseases of Ministry of Education of China, Key Laboratory for Disease Prevention and Control and Health Promotion of Shaanxi Province, School of Public Health, Health Science Center, Xi'an Jiaotong University, Xi'an, P. R. China; Key Laboratory of Trace Elements and Endemic Diseases of National Health and Family Planning Commission, Key Laboratory of Environment and Genes Related to Diseases of Ministry of Education of China, Key Laboratory for Disease Prevention and Control and Health Promotion of Shaanxi Province, School of Public Health, Health Science Center, Xi'an Jiaotong University, Xi'an, P. R. China; Key Laboratory of Trace Elements and Endemic Diseases of National Health and Family Planning Commission, Key Laboratory of Environment and Genes Related to Diseases of Ministry of Education of China, Key Laboratory for Disease Prevention and Control and Health Promotion of Shaanxi Province, School of Public Health, Health Science Center, Xi'an Jiaotong University, Xi'an, P. R. China; Key Laboratory of Trace Elements and Endemic Diseases of National Health and Family Planning Commission, Key Laboratory of Environment and Genes Related to Diseases of Ministry of Education of China, Key Laboratory for Disease Prevention and Control and Health Promotion of Shaanxi Province, School of Public Health, Health Science Center, Xi'an Jiaotong University, Xi'an, P. R. China; Key Laboratory of Trace Elements and Endemic Diseases of National Health and Family Planning Commission, Key Laboratory of Environment and Genes Related to Diseases of Ministry of Education of China, Key Laboratory for Disease Prevention and Control and Health Promotion of Shaanxi Province, School of Public Health, Health Science Center, Xi'an Jiaotong University, Xi'an, P. R. China; Key Laboratory of Trace Elements and Endemic Diseases of National Health and Family Planning Commission, Key Laboratory of Environment and Genes Related to Diseases of Ministry of Education of China, Key Laboratory for Disease Prevention and Control and Health Promotion of Shaanxi Province, School of Public Health, Health Science Center, Xi'an Jiaotong University, Xi'an, P. R. China; Key Laboratory of Trace Elements and Endemic Diseases of National Health and Family Planning Commission, Key Laboratory of Environment and Genes Related to Diseases of Ministry of Education of China, Key Laboratory for Disease Prevention and Control and Health Promotion of Shaanxi Province, School of Public Health, Health Science Center, Xi'an Jiaotong University, Xi'an, P. R. China; Key Laboratory of Trace Elements and Endemic Diseases of National Health and Family Planning Commission, Key Laboratory of Environment and Genes Related to Diseases of Ministry of Education of China, Key Laboratory for Disease Prevention and Control and Health Promotion of Shaanxi Province, School of Public Health, Health Science Center, Xi'an Jiaotong University, Xi'an, P. R. China; Key Laboratory of Trace Elements and Endemic Diseases of National Health and Family Planning Commission, Key Laboratory of Environment and Genes Related to Diseases of Ministry of Education of China, Key Laboratory for Disease Prevention and Control and Health Promotion of Shaanxi Province, School of Public Health, Health Science Center, Xi'an Jiaotong University, Xi'an, P. R. China; Key Laboratory of Trace Elements and Endemic Diseases of National Health and Family Planning Commission, Key Laboratory of Environment and Genes Related to Diseases of Ministry of Education of China, Key Laboratory for Disease Prevention and Control and Health Promotion of Shaanxi Province, School of Public Health, Health Science Center, Xi'an Jiaotong University, Xi'an, P. R. China; Key Laboratory of Trace Elements and Endemic Diseases of National Health and Family Planning Commission, Key Laboratory of Environment and Genes Related to Diseases of Ministry of Education of China, Key Laboratory for Disease Prevention and Control and Health Promotion of Shaanxi Province, School of Public Health, Health Science Center, Xi'an Jiaotong University, Xi'an, P. R. China; Key Laboratory of Trace Elements and Endemic Diseases of National Health and Family Planning Commission, Key Laboratory of Environment and Genes Related to Diseases of Ministry of Education of China, Key Laboratory for Disease Prevention and Control and Health Promotion of Shaanxi Province, School of Public Health, Health Science Center, Xi'an Jiaotong University, Xi'an, P. R. China; Key Laboratory of Trace Elements and Endemic Diseases of National Health and Family Planning Commission, Key Laboratory of Environment and Genes Related to Diseases of Ministry of Education of China, Key Laboratory for Disease Prevention and Control and Health Promotion of Shaanxi Province, School of Public Health, Health Science Center, Xi'an Jiaotong University, Xi'an, P. R. China

**Keywords:** polygenic and gene–environment interaction risk score (PGIRS), depression, cardiac function, lung function

## Abstract

**Background:**

Depression has been linked to an increased risk of cardiovascular and respiratory diseases; however, its impact on cardiac and lung function remains unclear, especially when accounting for potential gene–environment interactions.

**Methods:**

We developed a novel polygenic and gene–environment interaction risk score (PGIRS) integrating the major genetic effect and gene–environment interaction effect of depression-associated loci. The single nucleotide polymorphisms (SNPs) demonstrating major genetic effect or environmental interaction effect were obtained from genome-wide SNP association and SNP-environment interaction analyses of depression. We then calculated the depression PGIRS for non-depressed individuals, using smoking and alcohol consumption as environmental factors. Using linear regression analysis, we assessed the associations of PGIRS and conventional polygenic risk score (PRS) with lung function (*N* = 42 886) and cardiac function (*N* = 1791) in the subjects with or without exposing to smoking and alcohol drinking.

**Results:**

We detected significant associations of depression PGIRS with cardiac and lung function, contrary to conventional depression PRS. Among smokers, forced vital capacity exhibited a negative association with PGIRS (*β* = −0.037, FDR = 1.00 × 10^−8^), contrasting with no significant association with PRS (*β* = −0.002, FDR = 0.943). In drinkers, we observed a positive association between cardiac index with PGIRS (*β* = 0.088, FDR = 0.010), whereas no such association was found with PRS (*β* = 0.040, FDR = 0.265). Notably, in individuals who both smoked and drank, forced expiratory volume in 1-second demonstrated a negative association with PGIRS (*β* = −0.042, FDR = 6.30 × 10^−9^), but not with PRS (*β* = −0.003, FDR = 0.857).

**Conclusions:**

Our findings underscore the profound impact of depression on cardiac and lung function, highlighting the enhanced efficacy of considering gene–environment interactions in PRS-based studies.

## INTRODUCTION

Depression is a major public health problem worldwide, accounting for a significant cause of disability and disease burden among mental disorders [1]. It commonly co-occurs with cardiovascular and respiratory diseases, raising the risk of developing these pathologies and mortality rates [2–5]. Cardiac and lung functions are important indicators for predicting and reflecting cardiovascular and respiratory diseases. Left ventricular dysfunction, for instance, has been observed in individuals with depression post-cardiovascular diseases, holding crucial implications for cardiovascular prognosis [6]. Abnormal trajectories in lung function are associated with an increased susceptibility to respiratory diseases, early multimorbidity and premature mortality [7]. Explicitly assessing cardiac and lung function is helpful in identifying risk factors, thereby facilitating early prevention and treatment of the cardiovascular and respiratory diseases. Despite previous studies have demonstrated a link between depression and cardiovascular/ respiratory diseases, limited evidence exists regarding the relationship between depression with cardiac function in the general population, and the association between depression and lung function remains controversial [8, 9].

The polygenic risk score (PRS) emerges as a powerful tool for predicting an individual’s genetic susceptibility to complex traits and diseases [10]. Calculated by assigning weights to single nucleotide polymorphisms (SNPs) based on their effect sizes [11], PRS has garnered considerable attention in diverse fields, such as personalized genomic medicine [12] and disease risk prediction and prevention [13]. For instance, the schizophrenia PRS has successfully predicted cardiac structure in individuals without schizophrenia [14], and the PRS for coronary artery disease has shown potential in identifying individuals benefiting from intensive lifestyle modifications, imaging surveillance and early statin therapy [15]. Given the moderate heritability of depression as a complex, polygenic disorder [16], genome-wide PRS has been widely used to investigate the associations between genetic susceptibility of depression and multiple diseases in Phenome-wide association study (PheWAS) [17].

All organisms are the manifestation of encrypted genetic information in a specific environment [18]. Complex diseases are understood to result from a combination of genetic predisposition and environmental influences, with gene–environment interactions playing a crucial role in susceptibility and progression of diseases [19]. For example, the interaction between functional polymorphisms of the serotonin transporter gene (5-HTTLPR) and stressful life events significantly influences the risk of developing depression [20, 21], while in the absence of adverse life events, 5-HTTLPR polymorphisms do not modify the risk of depression [20]. Conventional PRS-based PheWAS applied genome-wide PRS to test the associations between different phenotypes without considering the contribution of gene–environment interactions. Combining PRS-based predictions with environmental exposures can improve the predictive power of phenotypic risk [22]. Our hypothesis posits that if depression shares genetic mechanisms with cardiac and lung function under specific environmental factors, polygenic risk of depression incorporating gene–environment interaction would be a more effective predictor than conventional PRS. Based on this, we developed a novel polygenic and gene–environment interaction risk score (PGIRS) by integrating major genetic effects and gene–environment interaction effects of genetic loci identified through genome-wide SNP association and SNP-environment interaction analysis. Unlike conventional PRS, PGIRS accounts not only for the major genetic effects of GWAS SNPs but also the interaction effects between genetic and environmental factors, potentially enhancing the predictive power of associations between diseases or traits.

In this study, we initially conducted genome-wide SNP association and SNP-environment interaction analysis for depression, incorporating smoking and drinking alcohol as environment factors. Subsequently, we computed PGIRS and conventional PRS for depression based on the SNPs identified in genome-wide association analysis among non-depression individuals. Finally, we analyzed the associations of depression PGIRS and PRS with cardiac and lung functions.

## METHODS

### Study sample and design

The study samples were from UK Biobank cohort (application 46478). UK Biobank is a large-scale population-based prospective study composing of biological samples and phenotype data from more than 500 000 people aged 40–69 years assessed between 2006 and 2010 in 22 assessment centers throughout the UK. The participants required to finish the self-completed touch screen questionnaires and brief computer-assisted interviews in the assessment visit. UK Biobank had obtained ethics approval from the North West Multi-center Research Ethics Committee (approval number: 11/NW/0382), and all participants have signed an informed consent, allowing UK Biobank to access their health-related records [23].

**Figure 1:**
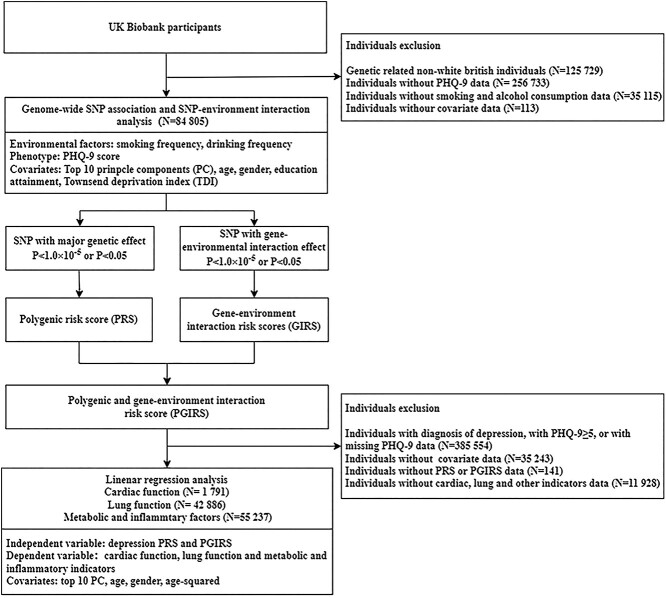
Flowchart of the study.

In this study, we conducted genome-wide SNP association and SNP–environment interaction analysis for depression, with Patient Health Questionnaire-9 (PHQ-9) score [24] as the measurement of depression, and smoking and drinking alcohol as environment factors. Subsequently, we computed PGIRS and conventional PRS for depression based on the SNPs identified in genome-wide association analysis. Finally, we investigated the associations of depression PGIRS and PRS with cardiac and lung functions, excluding individuals with a diagnosis of depression or positive symptoms of depression to distinguish PRS associations driven by the primary trait diagnosis from independent associations due to genetic risk [24]. The design of the whole study is shown in Figure 1.

### Definition of phenotypes

This study assessed various indicators related to cardiac and lung function. Cardiac function parameters included augmentation index, and measures of left ventricle (LV) volume and function, specifically encompassing cardiac output, LV ejection fraction, LV end-diastolic volume, LV end-systolic volume and LV stroke volume. Lung function was evaluated through metrics such as forced expiratory volume in 1 s (FEV1), forced vital capacity (FVC), the FEV1/FVC ratio and peak expiratory flow (PEF).

Moreover, metabolic and inflammatory indicators have been found to be associated with cardiac and lung functions, having potential role in crucial aspects of cardiac and lung health [25–28], and they were also intricately intertwined with onset and development of depression [29, 30]. Inflammatory states or metabolic abnormalities can co-occur in both mental disorders and cardiovascular or pulmonary diseases, which might mediate or regulate the association between them [31–35]. Thus, we explored the association of depression PRS and PGIRS with body mass index (BMI), waist to hip ratio (WHR), blood lipids and C-reaction protein (CRP) in the additional analysis. The details for phenotypes definition are shown in supplementary materials (see Supplementary Data available online at http://bib.oxfordjournals.org/).

### UK biobank genotyping, imputation and quality control

The genetic data from the UK Biobank included genotypes from 488 377 individuals assessed by two similar genotyping arrays: the Applied Biosystems UK BiLEVE Axiom Array and the Applied Biosystems UK Biobank Axiom [36]. IMPUTE4 was used for imputation, which was conducted in chunks of ~50 000 imputed markers with a 250 kb buffer region and on 5000 samples per compute job. Routine quality control was performed during sample and DNA extraction, as well as genotyping. To ensure the consistency of genotype calling, statistical tests were performed to identify poor quality markers due to batch effects, plate effects and deviation from Hardy–Weinberg equilibrium (HWE). Principal component (PC) analysis was used to account for population structure in both sample-based quality and marker control [36].

### Genome-wide SNP association and SNP–environment interaction analyses

We performed genome-wide SNP association and SNP–environment interaction analysis to identify genetic loci for depression. PLINK 2.0 was used for analysis based on generalized linear model: $Y={b}_0+{b}_gG+{b}_eE+{b}_{ge}G\cdotp E$, $Y$ represents the phenotype vector, $G$ represents genetic factor (the risk allele of the SNP), $E$ represents the environmental variable and $G\cdotp E$ represents the SNP–environment interaction [37]. ${b}_g$ represents the effect parameter for the risk allele of the SNP, ${b}_e$ represents the effect of environment variable and ${b}_{ge}$ represents the effect of SNP–environment interactions. The SNPs with low call rates <0.90, low HWE exact test *P*-value <0.001 or low minor allele frequencies <0.01 were excluded for quality control.

PHQ-9 score was used for quantitative measurement of depression. The daily smoking frequency and weekly drinking frequency were considered environmental factors. The PHQ-9 score was adjusted by age, gender, Townsend Deprivation Index, education attainment and top 10 PCs.

### Calculation of genetic risk for depression

The PRS and PGIRS of depression were assessed using the SNPs that demonstrated major genetic effect and interaction effect in genome-wide SNP association and SNP–environment interaction analysis. PLINK 2.0 was employed for analysis. Briefly, SNPs with major genetic effects were employed to compute the PRS, while SNPs displaying gene–environment interaction effects were utilized for calculating PGIRS, with a threshold set at *P* = 1.0 × 10^−5^. The SNPs were clumped using a window size of 250 kb and a threshold of *r*^2^ < 0.2. Additionally, sensitivity analysis was conducted using a threshold of *P* = 0.05.

The formula for PRS calculation is as follows:


$$ \mathrm{PRS}={\sum}_{i=1}^j{b}_{gi}{d}_i. $$

For each individual, $i$ ($i$=1,2,3…$j$) represents the number of SNPs with major genetic effect; ${b}_{gi}$ is the effect parameter for the risk allele of the $i$th SNP and ${d}_i$ is the dose (0 to 2) of the risk allele for the $i$th SNP.

The gene–environment interaction risk score (GIRS) was calculated based on the formula as following:


$$ \mathrm{GIRS}={\sum}_{k=1}^m{b}_{\mathrm{gek}}{f}_k\cdotp e. $$

For each individual, $k$ ($k$=1,2,3…$m$) represents the number of SNPs with gene–environment interaction effect; ${b}_{\mathrm{gek}}$ is the effect parameter of the interaction between risk allele of the $k$th SNP and environment factor, and ${f}_k$ is the dose (0–2) of the risk allele of $k$th SNP; $e$ represents the environment factor.

PGIRS is obtained by summing PRS and GIRS 


$$ \mathrm{PGIRS}=\mathrm{PRS}+\mathrm{GIRS}. $$

When considering gene–environment interactions of multiple exposures, the corresponding PGIRS was calculated by summing PRS and multiple GIRS. Let ‘a’ be weekly drinking frequency and ‘b’ be daily smoking frequency


$$ \mathrm{PGIRS}=\mathrm{PRS}+{\mathrm{GIRS}}_{\mathrm{a}}+{\mathrm{GIRS}}_{\mathrm{b}}. $$

### Statistical analysis

Standardized linear regression model was applied to investigate the impact of depression PRS and PGIRS on cardiac and lung function. The analysis was stratified based on the subjects’ smoking and alcohol consumption status. Briefly, analyzes were performed separately for smokers, non-smokers, drinkers, non-drinkers, individuals who both smoke and drink, and individuals who neither drink nor smoke. Age, gender, age-squared and the top 10 PCs were included as covariates. For smokers and non-smokers, the weekly drinking frequency was also considered as a covariate. For drinkers and non-drinkers, daily smoking frequency was adjusted. We also examined the association between depression PRS and PGIRS with metabolic and inflammatory indicators. The significance threshold was adjusted by false discovery rate (FDR) correction, and FDR <0.05 was considered significant.

For significant associations between depression genetic risk and cardiac and lung functions, we further investigated the mediating role of metabolic and inflammatory factors in theses associations, using PROCESS() function of ‘bruceR’ package. Additionally, we also performed K-fold cross validation (CV) analysis for significant associations detected in regression analysis to test the performance of the models, with *k* equal 5 in all CVs [38]. The ‘caret’ package was applied for CV analysis. All analysis was performed using R 4.1.0.

## RESULTS

### Sample characteristics

Among the 81 383 non-depressed individuals with depression PGIRS, conventional depression PRS and covariate data, a total of 1791, 42 886 and 55 237 participants with cardiac function, lung function and biochemical indicator data were included in the analysis (Tables 1 and S1, see Supplementary Data available online at http://bib.oxfordjournals.org/). Approximately 30% of individuals have ever smoked, 96% had a history of alcohol consumption and approximately 30% of individuals both smoked and drank alcohol. The cardiac and lung function phenotype had a roughly normal distribution (Figure S1, see Supplementary Data available online at http://bib.oxfordjournals.org/). Except for the skewed distribution of CRP, lipoprotein A and triglycerides, other metabolic and inflammatory indicators were approximately normal distribution (Figure S2, see Supplementary Data available online at http://bib.oxfordjournals.org/).

**Table 1:** Demographic characteristics of non-depressive individuals in the UK Biobank

		Cardiac function	Lung function	Metabolic and inflammatory indicators
Total *N*		1791	42 886	55 237
Gender (*N*)	Male	904	18 487	26 460
	Female	887	24 399	28 777
Age (Mean, SD)		56.43, 7.34	56.23, 7.49	56.42, 7.58
Smoking (*N*)	Yes	557	13 487	16 982
	No	1234	29 039	38 255
Drinking (*N*)	Yes	1744	41 509	53 144
	No	47	1377	2093

### Association between depression PGIRS, PRS and lung function

We observed that depression PGIRS was associated with multiple lung functions in the regression analysis, but most of lung functions were not associated with conventional depression PRS (Tables 2 and 3). For instance, in smoking individuals, we detected negative associations of depression PGIRS-smoke with forced expiratory volume in 1-second (FVE1) (*β* = −0.040, FDR = 5.02 × 10^−9^) and FVC (*β* = −0.037, FDR = 1.00 × 10^−8^), but no significant associations were observed for conventional PRS with FVE1 (*β* = −0.003, FDR = 0.847) and FVC (*β* = −0.002, FDR = 0.943). For drinkers, FVE1/FVC was found to be negatively associated with depression PGIRS-alcohol (*β* = −0.029, FDR = 6.62 × 10^−7^), but not associated with PRS (*β* = −0.005, FDR = 0.635). In individuals who both smoke and drink, we found that FVE1 was negatively associated with PGIRS-alcohol (*β* = −0.016, FDR = 0.046), PGIRS-smoke (*β* = −0.040, FDR = 3.70 × 10^−9^) and PGIRS-smoke-alcohol (*β* = −0.042, FDR = 6.30 × 10^−9^). However, we did not detect significant association between depression PRS and FVE1 (*β* = −0.003, FDR = 0.857). The sensitivity analysis yielded consistent results with the main analysis, despite we detected associations of PRS with FVE1 and FVC for individuals who neither smoke nor drink alcohol in the main analysis, but the associations were not replicated in sensitivity analysis (Tables S2, S3 and S4).

**Table 2:** Association between polygenic risk of depression with cardiac and lung function stratified by smoking or drinking alcohol

	Non-smokers		Smokers				Non-drinkers		Drinkers			
	PRS		PRS		PGIRS-smoke		PRS		PRS		PGIRS-alcohol	
Traits	Beta	FDR	Beta	FDR	Beta	FDR	Beta	FDR	Beta	FDR	Beta	FDR
PEF	−0.007	0.325	−0.006	0.685	−0.021	0.012^*^	0.005	0.943	−0.007	0.217	−0.005	0.589
FVE1	−0.006	0.385	−0.003	0.847	−0.040	5.02 × 10^–9**^	0.043	0.066	−0.006	0.247	−0.003	0.737
FVC	−0.004	0.622	−0.002	0.943	−0.037	1.00 × 10^–8**^	0.041	0.077	−0.004	0.542	0.006	0.338
FVE1/FVC	−0.004	0.725	−0.004	0.860	−0.016	0.225	0.007	0.945	−0.005	0.635	−0.029	6.62 × 10^–7**^
Augmentation index	−0.025	0.651	0.030	0.724	0.077	0.171	−0.276	0.246	−0.005	0.948	−0.006	0.948
Cardiac index	0.043	0.325	0.029	0.777	0.006	0.959	−0.087	0.860	0.040	0.265	0.088	0.010^*^
LV ejection fraction	0.002	0.968	0.044	0.581	0.009	0.948	−0.033	0.945	0.013	0.796	−0.014	0.823
LV end diastolic volume	0.019	0.685	−0.031	0.74	−0.027	0.801	0.016	0.954	0.005	0.945	0.040	0.354
LV end systolic volume	0.009	0.896	−0.047	0.589	−0.035	0.716	0.031	0.820	−0.007	0.920	0.024	0.674
LV stroke volume	0.040	0.354	0.026	0.809	0.009	0.953	−0.026	0.954	0.035	0.351	0.064	0.084

^**^FDR ≤ 0.001, ^*^FDR ≤ 0.05.

**Table 3:** Association between polygenic risk of depression with cardiac and lung function stratified by smoking and drinking alcohol

	Individuals who neither smoke nor drink		Individuals who both smoke and drink							
	PRS		PRS		PGIRS-alcohol		PGIRS-smoke		PGIRS-smoke-alcohol	
	Beta	FDR	Beta	FDR	Beta	FDR	Beta	FDR	Beta	FDR
PEF	0.013	0.797	−0.005	0.716	−0.015	0.116	−0.022	0.009^*^	−0.026	2.09 × 10^–3**^
FVE1	0.053	0.026	−0.003	0.857	−0.016	0.046^*^	−0.040	3.70 × 10^–9**^	−0.042	6.30 × 10^–9**^
FVC	0.057	0.013	−0.001	0.961	−0.002	0.945	−0.036	2.61 × 10^–8**^	−0.028	1.53 × 10^–4**^
FVE1/FVC	−0.009	0.909	−0.007	0.725	−0.048	2.74 × 10^–7**^	−0.021	0.081	−0.054	7.56 × 10^–8**^
Augmentation index	−0.269	0.280	0.031	0.716	0.021	0.860	0.078	0.164	0.065	0.386
Cardiac index	−0.093	0.860	0.028	0.795	0.060	0.530	0.008	0.954	0.040	0.716
LV ejection fraction	−0.025	0.954	0.044	0.577	0.023	0.829	0.011	0.943	0.005	0.968
LV end diastolic volume	0.018	0.948	−0.033	0.724	2.60 × 10^−4^	0.995	−0.031	0.740	−0.009	0.954
LV end systolic volume	0.033	0.811	−0.048	0.589	−0.012	0.945	−0.040	0.660	−0.017	0.904
LV stroke volume	−0.024	0.957	0.024	0.824	0.034	0.768	0.007	0.957	0.020	0.889

^**^FDR ≤ 0.001, ^*^FDR ≤ 0.05.

### Association between depression PGIRS, PRS and cardiac function

For cardiac functions, we identified positive association between PGIRS-alcohol and cardiac index in drinkers (*β* = 0.088, FDR = 0.010) (Tables 2 and 3). No significant association was found between cardiac index and conventional PRS (*β* = 0.040, FDR = 0.265). The remaining cardiac functions were not significantly associated with either PGIRS or PRS. The results of the sensitivity analysis were generally consistent with the findings in main analysis (Tables S2, S3 and S4).

### Association between depression PGIRS, PRS and metabolic, inflammatory indicators

**Figure 2:**
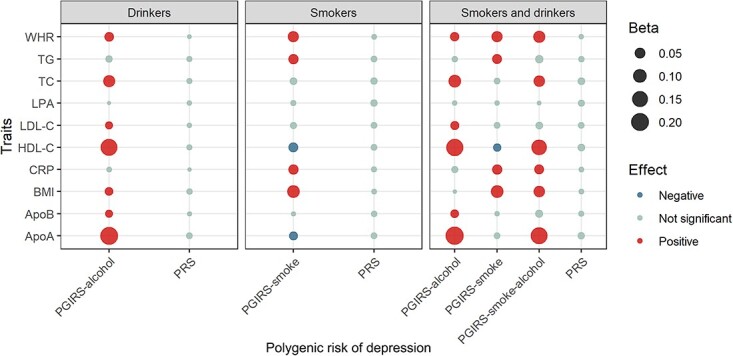
Association between polygenic risk of depression with metabolic and inflammatory indicators. ^*^The bubble plot shows the association between PGIRS of depression and PRS with metabolic and inflammatory indicators. The size of the bubble represents the size of the association effect. Abbreviations: low-density lipoprotein cholesterol, LDL-C; high-density lipoprotein cholesterol, HDL-C; triglycerides, TG; total cholesterol, TC; Apolipoprotein B, ApoB; Apolipoprotein A, ApoA; Lipoprotein(a), LPA.

For individuals smoking or drinking alcohol, we observed that depression PGIRS was significantly associated with multiple metabolic and inflammatory indicators. However, none indicator was observed to be associated with conventional depression PRS (Figure 2). In drinkers, BMI (*β* = 0.021, FDR = 5.08 × 10^−6^) and low-density lipoprotein cholesterol (LDL-C) (*β* = 0.015, FDR = 7.76 × 10^−3^) were found to be positively associated with PGIRS-alcohol, but not associated with PRS (*β* = 0.005, FDR = 0.447 for BMI; *β* = 0.002, FDR = 0.870 for LDL-C). In smokers, we found that PGIRS-smoke was negatively associated with high-density lipoprotein cholesterol (HDL-C) (*β* = −0.035, FDR = 2.05 × 10^−6^), and positively related to CRP (*β* = 0.039, FDR = 2.74 × 10^−7^), but we did not detect significant associations of PRS with HDL-C (*β* = 0.008, FDR = 0.556) and CRP (*β* = 0.004, FDR = 0.801). In individuals who both smoke and drink alcohol, we found that HDL-C was significantly associated with PGIRS-smoke (*β* = −0.019, FDR = 0.033), PGIRS-alcohol (*β* = 0.187, FDR = 1.78 × 10^−159^) and PGIRS-smoke-alcohol (*β* = 0.142, FDR = 5.00 × 10^−80^). No significant association was observed between HDL-C and conventional PRS (*β* = 0.013, FDR = 0.201). The results of the sensitivity analysis were consistent with the findings in the main analysis (Tables S2, S3 and S4).

### Mediation analysis

We conducted mediation analysis to explore the mediating role of inflammatory or metabolic factors in the association between depression PGIRS and cardiac and lung functions, and observed several significant mediating effects (Table S5, see Supplementary Data available online at http://bib.oxfordjournals.org/). For instance, in smoking individuals, CRP positively mediated the negative relationship between PGIRS-smoke and FVC (*β*_Indirect_ = −0.003, *β*_Total_ = −0.034). In drinking individuals, HDL-C positively mediated the positive relationship between PGIRS-alcohol and cardiac index (*β*_Indirect_ = 0.014, *β*_Total_ = 0.112). For individuals who both smoke and drink alcohol, we observed that WHR showed positive mediation effect for the negative association of PGIRS-smoke-alcohol and FVE1 (*β*_Indirect_ = −0.011, *β*_Total_ = −0.045).

### Model evaluation results

K-fold CV analysis was performed for significant associations detected in regression analysis to test the performance of the models (Table S6, see Supplementary Data available online at http://bib.oxfordjournals.org/). The root mean squared error of models ranges from 0.094 to 0.786, and Mean absolute error ranges from 0.062 to 0.628. The R-squared (*R*^2^) ranges from 0.139 to 0.991, with 50% of the models having an *R*^2^ greater than 0.8 and 80% of the models having an *R*^2^ greater than 0.5.

## DISCUSSION

In this study, we constructed PGIRS for depression, integrating both major genetic effects and gene–environment interaction effects of genetic loci identifying by genome-wide SNP association and SNP–environment interaction analysis. We explored the effects of PGIRS and conventional PRS on cardiac and lung functions, as well as metabolic and inflammatory indicators in non-depressed individuals. We observed significant associations between PGIRS and multiple traits, whereas most of these indicators did not show associations with PRS. Our findings highlight the connections between depression and cardiac/lung functions, suggesting shared genetic mechanisms regulated by environmental factors.

We detected negative associations between depression PGIRS and lung functions among smokers and drinkers. In a prospective study, persistent depressive symptoms were significantly associated with accelerated declines in FVC in male smokers, with no observed relationship in male non-smokers [39]. Similarly, an inverse association between depressive symptoms and lung function was found in healthy adults, especially among those with a heavy smoking history [40]. These findings imply an important role of smoking in the association between depression and respiratory function.

While previous studies reported lower cardiac input in the depressed subjects compared with non-depressed individuals [41], our study revealed a positive association between cardiac index and depression PGIRS-alcohol, with no significant association observed with depression PRS. Alcoholic drink was found to increase the cardiac output [42]. A recent study also noted that light or moderate alcohol consumption was associated with lower risk of major adverse cardiovascular events, suggesting the role of alcohol consumption in lowering activity of a stress-related brain network known for its association with cardiovascular disease [43]. We speculate that the positive correlation between depression PGIRS and cardiac index may be attributed to the interaction between genetic factors and alcohol consumption, which requires further investigation.

We also found several metabolic and inflammatory indicators associated with PGIRS, such as LDL-C and CRP. In MDD patients, smoking was associated with higher LDL-C levels [44]. Previous research observed an association between PRS and CRP levels in depression, attributing it to a genetic contribution to increased inflammation influenced by dietary and smoking habits [45]. Moreover, we observed multiple metabolic and inflammatory indicators significantly mediated the association of depression PGIRS and cardiac and lung function, indicating that chronic inflammation and metabolic abnormalities may be common underlying pathophysiological processes in depression and cardiopulmonary dysfunction [31–35].

The practical utility of the PRS was found to be limited, despite its ability to capture stable traits with a normal distribution in the general population [11]. Gene–environment interaction has emerged as a significant factor in the etiology and development of mental disorders [20, 21]. The conventional PRS, which solely considers genetic effects, has inherent limitations when it comes to accurately predicting disease risk. The development of a single disease is not guaranteed solely by the inheritance of a genetic predisposition; instead, it relies on exposure to specific environmental triggers [46]. In light of these complexities, the PGIRS we developed offers a comprehensive assessment of an individual’s genetic susceptibility when considering specific environmental factors. In this study, the calculation of depression PGIRS and traditional PRS involves two key parameters: beta and the threshold for SNP inclusion. The beta values for calculations of depression PGIRS and traditional PRS were derived from the results of genome-wide SNP association and SNP–environment interaction analysis, also called genome-wide gene–environment interaction study, which has been widely applied to identify genetic variants for complex diseases [47–52]. For the threshold of SNP inclusion, we used the genome-wide suggestive significance level (*P* = 1.0 × 10^−5^) [53] for main analysis and applied nominal significance level (*P* = 0.05) [14], which is milder, for sensitivity analysis. We observed fundamentally consistent associations in analyzes using two thresholds. Moreover, the CV results indicate that the models perform satisfactorily in explaining the variability of the dependent variable [54, 55]. Specifically, half of the models demonstrate a high level of explanatory power with an *R*^2^ exceeding 0.8, while 80% of the models perform well at least at a moderate level (*R*^2^ greater than 0.5). This implies that these models are relatively accurate in capturing the variations of the dependent variable.

Previous studies commonly employed PRS-based phenotype association studies to detect both shared and distinct genetic associations between diseases and traits [56]. However, these approaches primarily capture simple associations between genetic loci and diseases, often overlooking shared susceptibility genes that may play a crucial role in specific environmental contexts. In this study, we constructed the PGIRS with a specific focus on smoking and drinking alcohol as shared environmental risk factors between depression with cardiac and lung function [57, 58]. We found that PGIRS incorporating major genetic effect and gene–environment interactions, rather than PRS, affect cardiac and lung functions in a manner associated with increased risk of respiratory and cardiac diseases. Our findings suggest that the shared gene–environment interactions may involve in depression and cardiopulmonary disease, supporting the argument for regular cardiac and lung screening in depression patients under specific environmental exposures, such as smoking and alcohol drinking. Hence, we argue that shared genetic variations among diseases may exert their effects in response to specific environmental exposures, that is, some genetic associations between diseases are attributed to common gene–environment interactions. Considering the regulation of the environment factors on genes is helpful in exploring genetic associations between various diseases and traits, providing more accurate risk predictions across diseases and phenotypes. In clinical applications and individualized interventions, environmental factors are easier to identify and control than genetic factors. Therefore, constructing PGIRS by evaluating the combined role of genetic and gene–environment interaction effect is meaningful for implementing targeted interventions and improvements in disease prevention and clinical management.

To the best of our knowledge, this study is the first to develop PGIRS, evaluating polygenic risk for complex traits by incorporating the major genetic effect and gene-interaction effect of SNPs. PGIRS considers the influence of environmental factors on genetic variants, allowing for a more comprehensive assessment of cumulative genetic disease risk. This approach surpasses conventional PRS in capturing the impact of environmental exposures on the genetics. Additionally, we identified multiple significant associations between PGIRS of depression and cardiac/lung function, as well as metabolic and inflammatory biochemical indicators. In contrast to PRS-based PheWAS, our approach offers the advantage of incorporating gene–environment interactions in elucidating the interrelationships between various traits within the context of specific environmental exposures. This comprehensive approach goes beyond solely assessing major genetic associations and allows for a more nuanced understanding of how gene–environment interactions contribute to the observed trait associations. Compared with conventional PRS, PGIRS exhibits greater predictive power in capturing associations across diverse diseases and traits. It should be note that utilizing an independent validation set can provide a more unbiased estimation of model performance. However, at present, we cannot obtain a suitable independent dataset containing both genotype and phenotype data for validation analysis. In future research, we will collect large-scale independent data to verify our findings and performed cell and animal experiments to explore the biological mechanism.

In summary, we developed a novel polygenic risk assessment index, PGIRS, which considers major genetic effects as well as gene–environment interactions. We identified significant associations of depression PGIRS with cardiac and lung function, whereas no significant associations were observed for PRS. Our findings indicate the existence of shared gene–environment interactions between depression and cardiac, lung function. We provide new insights into the exploration of genetic associations between diseases and traits, highlighting the potential of shared genetic loci regulated by characteristic environment factors, which have significant implications for individualized disease risk prediction and assessment in clinical settings.

**Key Points**

- The polygenic and gene–environment interaction risk score (PGIRS) is a novel polygenic risk assessment index that takes into account both major genetic effects and gene–environment interactions.
- Significant associations were found between PGIRS for depression and cardiopulmonary function, while no significant associations were observed for the polygenic risk score (PRS).
- PGIRS demonstrates greater efficacy in capturing associations across diverse traits when compared with PRS.
- Shared gene–environment interactions may be present between depression and cardiopulmonary function.

## Supplementary Material

Supplementary_materials(2)_bbae070

## Data Availability

The list of SNP detected in Genome-wide SNP association and SNP-environment interaction analyses is available in figshare database (https://figshare.com/articles/dataset/SNP_list_of_Genomewide_association_analysis_P_0_05_/25011698).

## FUNDINGS

Natural Science Basic Research Plan in Shaanxi Province of China (2021JCW-08); Fundamental Research Funds for the Central Universities (11913223000009).

## Author Biographies

**Chuyu Pan** is currently working as a doctor student at School of Public Health, Xi’an Jiaotong University.

**Bolun Cheng** is a postdoctoral researcher at the Key Laboratory of Trace Elements and Endemic Diseases of National Health Commission of the People’s Republic of China, School of Public Health, Health Science Center, Xi’an Jiaotong University.

**Xiaoyue Qin** is currently working as a postgraduate student at School of Public Health, Xi’an Jiaotong University.

**Shiqiang Cheng** is currently working as a doctor student at School of Public Health, Xi’an Jiaotong University.

**Li Liu** is currently working as a doctor student at School of Public Health, Xi’an Jiaotong University.

**Xuena Yang** is currently working as a doctor student at School of Public Health, Xi’an Jiaotong University.

**Peilin Meng** is currently working as a doctor student at School of Public Health, Xi’an Jiaotong University.

**Na Zhang** is currently working as a postgraduate student at School of Public Health, Xi’an Jiaotong University.

**Dan He** is currently working as a doctor student at School of Public Health, Xi’an Jiaotong University.

**Qingqing Cai** is currently working as a postgraduate student at School of Public Health, Xi’an Jiaotong University.

**Wenming Wei** is currently working as a doctor student at School of Public Health, Xi’an Jiaotong University.

**Jingni Hui** is currently working as a doctor student at School of Public Health, Xi’an Jiaotong University.

**Yan Wen** is an associate professor at the Key Laboratory of Trace Elements and Endemic Diseases of National Health Commission of the People’s Republic of China, School of Public Health, Health Science Center, Xi’an Jiaotong University.

**Yumeng Jia** is an associate professor at the Key Laboratory of Trace Elements and Endemic Diseases of National Health Commission of the People’s Republic of China, School of Public Health, Health Science Center, Xi’an Jiaotong University.

**Huan Liu** is a postdoctoral researcher at the Key Laboratory of Trace Elements and Endemic Diseases of National Health Commission of the People’s Republic of China, School of Public Health, Health Science Center, Xi’an Jiaotong University.

**Feng Zhang** is a professor at the Key Laboratory of Trace Elements and Endemic Diseases of National Health and Family Planning Commission, Key Laboratory of Environment and Genes Related to Diseases of Ministry of Education of China, Key Laboratory for Disease Prevention and Control and Health Promotion of Shaanxi Province, School of Public Health, Health Science Center, Xi'an Jiaotong University.
