# Supplementary material for: Enhanced polygenic risk score incorporating gene–environment interaction suggests the association of major depressive disorder with cardiac and lung function
Source: Brief Bioinform. 2024 Mar 2;25(2):bbae070. doi: 10.1093/bib/bbae070 (PMC11648690; doi:10.1093/bib/bbae070)
Supplement: Supplementary_materials(2)_bbae070 [file supplementary_materials(2)_bbae070.pdf]

Supplementary Materials

Supplementary Methods

Definition of phenotypes in the UK Biobank

Depression

Patient Health Questionnaire-9 (PHQ-9) [1] is a classification algorithm with a total score (0-27) used to screen for and measure depression severity, focusing on nine depressive symptoms and signs (as detailed below: Little interest or pleasure in doing things (UKB: 20514), Feeling down, depressed, or hopeless (UKB:20510, Trouble sleeping(UKB: 20517), Feeling tired (UKB: 20519), Poor appetite or overeating (UKB: 20511), Feeling bad about yourself (UKB: 20507), Trouble concentrating (UKB: 20508), Moving or speaking slowly or fidgety or restless (UKB: 20518), Thoughts that you would be better off dead (UKB: 20513)). In order to meet the 0-3 score for each item of PHQ, the 9 symptom scores (1-4) of our team UK were all reduced by 1 point, which was then added up. Additionally, to distinguish PRS associations driven by the primary trait diagnosis from independent associations due to genetic risk[25], we excluded individuals with a diagnosis of depression or positive symptoms of depression when investigating the association of depression polygenic risk with cardiac and lung functions. The diagnosis of depression was defined based on the International Classification of Disease version 10 (ICD-10), coding as F32-F33. The individuals with a PHQ-9 score ≥ 5 were considered to have positive depressive symptoms.

Cardiac Function

The volume and function of the left ventricle (LV) and augmentation index were used as the index of cardiac function. The LV size and function composed cardiac output, LV ejection

fraction, LV end diastolic volume, LV end systolic volume, and LV stroke volume. The LV size and function were measured by cardiovascular magnetic resonance (CMR) in the UK Biobank. The full CMR protocol and rationale have been described in detail previously[2]. All LV phenotypes were indexed to body surface area. The augmentation index was measured during pulse wave analysis and calculated by dividing the central augmentation pressure by the peripheral pulse pressure, expressing as percentage.

Lung function

Lung function were measured by forced expiratory volume in 1-second (FEV1), forced vital capacity (FVC), FEV1/FVC ratio and peak expiratory flow (PEF). The spirometry in the UK Biobank was assessed by nurses or healthcare technicians trained in spirometry following a standard protocol and using a Vitalograph Pneumotrac 6800 spirometer. According to the criteria recommended by the American Thoracic Society/European Respiratory Society (ATS/ERS) spirometer standardization working group, spirograms with at least two blows, a good end-blown quality, as well as reproducible FEV1 and FVC within 150 mL were considered best quality[3, 4]. The individuals with the best quality spirometry were retained in the analysis.

Metabolic and Inflammation indicators

Considering the impact of physiological factors, such as metabolic and inflammation factors, on cardiac and lung function[5], we also included body mass index (BMI), waist-hip ratio (WHR), blood lipids and C-reaction protein (CRP) in the analysis. The blood lipids contains low-density lipoprotein cholesterol (LDL-C), high-density lipoprotein cholesterol (HDL-C), triglycerides (TG), total cholesterol (TC), Apolipoprotein A (ApoA), Apolipoprotein B (ApoB) and Lipoprotein(a) (LPA). The blood biochemistry biomarkers were measured by enzymatic

protective selection analysis on a Beckman Coulter AU5800.

Daily smoking frequency

The daily smoking frequency was defined according to the ever-smoking status and the maximum number of reported past or current cigarettes consumed per day[6]. Briefly, we coded smoking frequency as 0 if ever-smoking status was also 0, otherwise, we used the maximum number of reported past or current cigarettes (or pipes/cigars) consumed per day.

Weekly drinking frequency

The weekly drinking frequency was constructed from responses to a sequence of questions[6]. The participants were firstly asked how often they drink alcohol, and response options include 1) daily or almost daily; 2) three or four times per week; 3) once or twice per week; 4) one to three times per month; 5) special occasions only; and 6) never. The individuals who reported drinking once per week or more were asked how many glasses of various types of alcoholic beverages they consume per week. We used the sum of all alcoholic drinks per week as the weekly drinking alcohol frequency for these individuals. The individuals who reported drinking less than once per week (one to three times per month or on special occasions only) were asked how many glasses of various types of alcoholic beverages they consume per month. For these participants, we added the total number of drinks per month and divided by 4 to arrive at an approximated number of alcoholic drinks per week. Individuals who reported never drinking were coded as 0.

Reference

1. Kroenke K, Spitzer RL, Williams JB, Löwe B. The Patient Health Questionnaire Somatic, Anxiety, and Depressive Symptom Scales: a systematic review. Gen Hosp Psychiatry. 2010;32(4):345-59.
2. Petersen SE, Matthews PM, Francis JM, Robson MD, Zemrak F, Boubertakh R, et al. UK Biobank's cardiovascular magnetic resonance protocol. J Cardiovasc Magn Reson. 2016;18:8.
3. Gupta RP, Strachan DP. Ventilatory function as a predictor of mortality in lifelong non-smokers: evidence from large British cohort studies. BMJ Open. 2017;7(7):e015381.
4. Miller MR, Crapo R, Hankinson J, Brusasco V, Burgos F, Casaburi R, et al. General considerations for lung function testing. Eur Respir J. 2005;26(1):153-61.
5. Wielscher M, Amaral AFS, van der Plaat D, Wain LV, Sebert S, Mosen-Ansorena D, et al. Genetic correlation and causal relationships between cardio-metabolic traits and lung function impairment. Genome Med. 2021;13(1):104.
6. Karlsson Linnér R, Biroli P, Kong E, Meddens SFW, Wedow R, Fontana MA, et al. Genome-wide association analyses of risk tolerance and risky behaviors in over 1 million individuals identify hundreds of loci and shared genetic influences. Nat Genet. 2019;51(2):245-57.

Supplementary Figures

Figure S1. The distribution of cardiac and lung function for UK Biobank participants.

Figure S2. The distribution of metabolic and inflammatory indicators for UK Biobank participants.

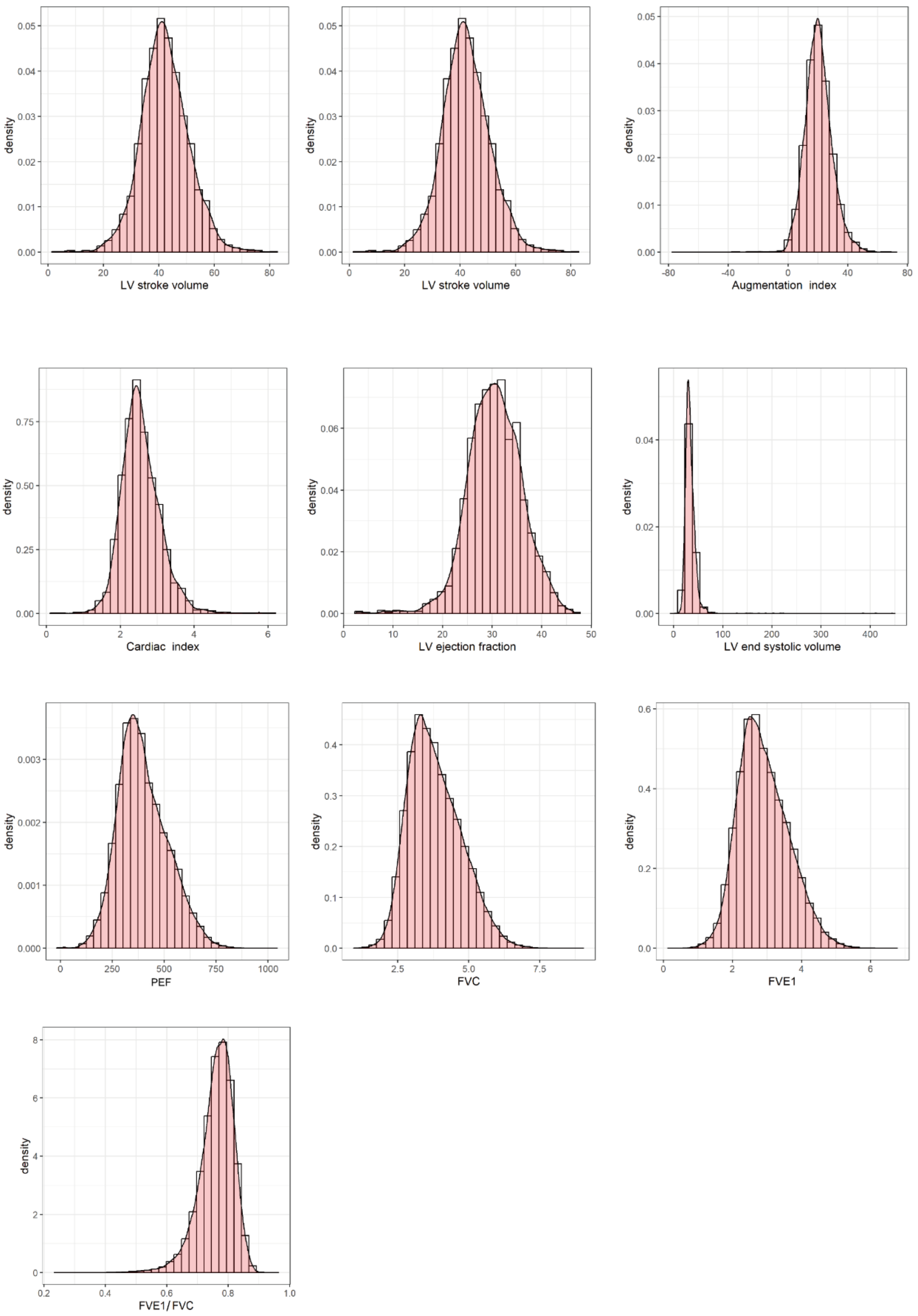

Figure S1. The distribution of cardiac and lung function for UK Biobank participants.

*Abbreviations: peak expiratory flow, PEF; forced expiratory volume in 1-second, FVE1; forced vital capacity, FVC.

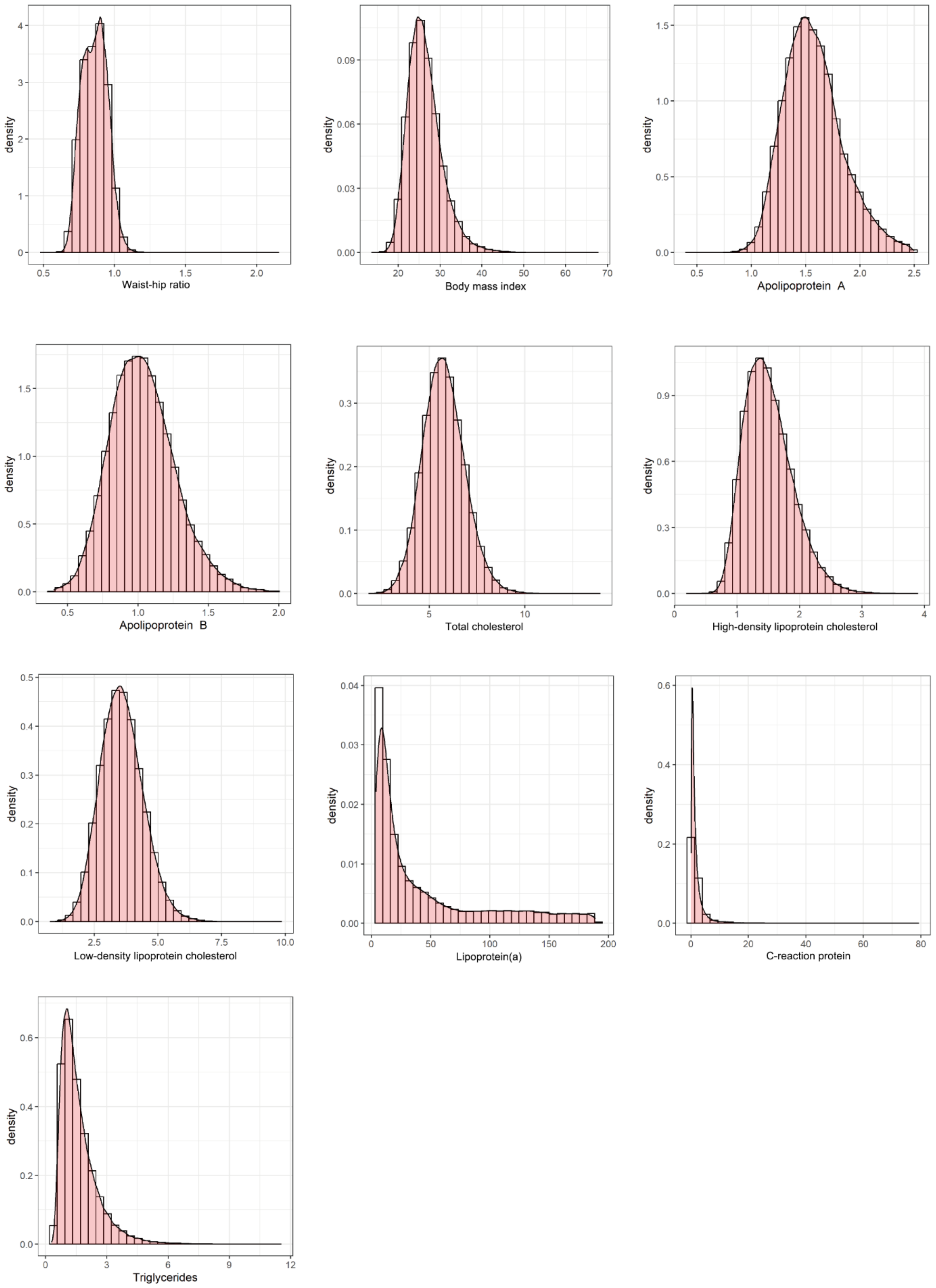

Figure S2. The distribution of metabolic and inflammatory indicators for UK Biobank participants.

Supplementary Tables

Table S1. Distribution characteristics of depression PRS and PGIRS.

Table S2. Associations of depression polygenic risk with cardiac function, lung function, and metabolic and inflammatory indicators in smoking& alcohol subgroups.

Table S3. Associations of depression polygenic risk with cardiac function, lung function, and metabolic and inflammatory indicators in smoking subgroups.

Table S4. Associations of depression polygenic risk with cardiac function, lung function, and metabolic and inflammatory indicators in alcohol subgroups.

Table S5. Significant results of mediation analysis.

Table S6. K-fold Cross validation Results.

Table S1. Distribution characteristics of depression PRS and PGIRS.

Threshold	Polygenic risk of depression	Cardiac Function	Lung Function	Metabolic and Inflammatory Indicators
1.00E-05	PRS	-0.0019±0.0041	-0.0017±0.0042	-0.0018±0.0041
	PGIRS-smoke	0.0006±0.0062	0.0008±0.0064	0.0007±0.0063
	PGIRS-alcohol	0.0046±0.0067	0.0046±0.0071	0.0045±0.0071
	PGIRS-smoke-alcohol	0.0071±0.0089	0.0072±0.0093	0.007±0.0093
0.05	PRS	-0.0048±0.0015	-0.0048±0.0015	-0.0048±0.0015
	PGIRS-smoke	-0.0004±0.0039	-0.0005±0.0041	-0.0005±0.0041
	PGIRS-alcohol	-0.0026±0.0042	-0.0026±0.0042	-0.0027±0.0042
	PGIRS-smoke-alcohol	0.0018±0.0061	0.0018±0.0064	0.0016±0.0064

***Abbreviations:** Polygenic risk score, PRS; Polygenic and gene-environment interaction risk scores, PGIRS.

Table S2. Associations of depression polygenic risk with cardiac function, lung function, and metabolic and inflammatory indicators in smoking&alcohol subgroups.

	Individuals who neither drink nor smoke				Individuals who both drink and smoke															
**P=1E-5**	**PRS**				**PRS**				**PGIRS-alcohol**				**PGIRS-smoke**				**PGIRS-smoke-alcohol**			
**Traits**	**Beta**	**SE**	**P**	**P_{FDR}**	**Beta**	**SE**	**P**	**P_{FDR}**	**Beta**	**SE**	**P**	**P_{FDR}**	**Beta**	**SE**	**P**	**P_{FDR}**	**Beta**	**SE**	**P**	**P_{FDR}**
PEF	0.013	0.020	0.520	0.797	-0.005	0.007	0.404	0.716	-0.015	0.007	0.029	0.116	-0.022	0.007	0.002	0.009	-0.026	0.007	3.42E-04	2.09E-03
FVE1	0.053	0.019	0.005	0.026	-0.003	0.006	0.610	0.857	-0.016	0.006	0.010	0.046	-0.040	0.006	3.61E-10	3.70E-09	-0.042	0.007	6.44E-10	6.30E-09
FVC	0.057	0.019	0.003	0.013	-0.001	0.006	0.896	0.961	-0.002	0.006	0.803	0.945	-0.036	0.006	2.91E-09	2.61E-08	-0.028	0.007	2.36E-05	1.53E-04
FVE1/FVC	-0.009	0.027	0.723	0.909	-0.007	0.008	0.433	0.725	-0.048	0.009	3.42E-08	2.74E-07	-0.021	0.009	0.019	0.081	-0.054	0.009	8.76E-09	7.56E-08
BMI	-0.024	0.023	0.302	0.629	0.003	0.007	0.669	0.887	2.49E-04	0.007	0.971	0.986	0.081	0.007	2.89E-32	6.06E-31	0.058	0.007	1.35E-15	1.65E-14
WHR	-0.004	0.018	0.808	0.945	-0.003	0.005	0.640	0.867	0.029	0.006	1.07E-07	7.86E-07	0.055	0.006	4.84E-23	7.89E-22	0.067	0.006	8.91E-30	1.63E-28
ApoA	0.003	0.019	0.877	0.957	0.012	0.007	0.090	0.290	0.215	0.007	1.91E-194	1.20E-192	-0.007	0.007	0.326	0.648	0.175	0.008	7.23E-113	3.18E-111
ApoB	-0.010	0.023	0.670	0.887	0.005	0.008	0.518	0.797	0.021	0.008	0.008	0.039	0.002	0.008	0.797	0.945	0.017	0.008	4.17E-02	0.157
TC	-0.010	0.023	0.644	0.869	0.013	0.007	0.078	0.264	0.082	0.007	8.38E-28	1.47E-26	-0.008	0.007	0.316	0.637	0.059	0.008	1.26E-13	1.46E-12
CRP	-0.033	0.021	0.114	0.349	0.004	0.007	0.600	0.849	0.010	0.007	0.164	0.446	0.039	0.007	3.12E-08	2.54E-07	0.036	0.008	2.36E-06	1.57E-05
HDL-C	0.004	0.019	0.822	0.948	0.013	0.007	0.055	0.201	0.187	0.007	3.24E-161	1.78E-159	-0.019	0.007	0.007	0.033	0.142	0.007	1.36E-81	5.00E-80
LPA	-0.021	0.022	0.350	0.664	0.009	0.008	0.254	0.589	0.002	0.008	0.777	0.943	0.002	0.008	0.825	0.948	-0.001	0.009	0.916	0.968
TG	-0.007	0.021	0.744	0.927	0.006	0.007	0.381	0.693	-0.006	0.007	0.422	0.724	0.036	0.007	1.37E-06	9.41E-06	0.018	0.008	0.021	0.088
LDL-C	-0.013	0.023	0.571	0.827	0.008	0.007	0.282	0.612	0.026	0.008	0.001	4.07E-03	-0.008	0.008	0.294	0.622	0.013	0.008	0.118	0.354
Augmentation index	-0.269	0.152	0.086	0.280	0.031	0.038	0.408	0.716	0.021	0.042	0.627	0.860	0.078	0.038	0.044	0.164	0.065	0.043	0.138	0.386
Cardiac index	-0.093	0.183	0.615	0.860	0.028	0.042	0.511	0.795	0.060	0.047	0.202	0.530	0.008	0.043	0.858	0.954	0.040	0.048	0.404	0.716
LV ejection fraction	-0.025	0.127	0.847	0.954	0.044	0.037	0.231	0.577	0.023	0.041	0.575	0.829	0.011	0.037	0.778	0.943	0.005	0.042	0.911	0.968
LV end diastolic volume	0.018	0.082	0.830	0.948	-0.033	0.041	0.424	0.724	2.67E-04	0.046	0.995	0.995	-0.031	0.042	0.455	0.740	-0.009	0.047	0.852	0.954
LV end systolic volume	0.033	0.053	0.542	0.811	-0.048	0.042	0.243	0.589	-0.012	0.046	0.804	0.945	-0.040	0.042	0.345	0.660	-0.017	0.048	0.715	0.904
LV stroke volume	-0.024	0.152	0.875	0.957	0.024	0.043	0.565	0.824	0.034	0.047	0.480	0.768	0.007	0.043	0.876	0.957	0.020	0.049	0.682	0.889
**P=0.05**																				
PEF	-0.022	0.019	0.259	0.589	0.010	0.007	0.123	0.361	-0.011	0.007	0.125	0.364	-0.022	0.008	0.005	0.026	-0.027	0.008	0.001	0.005
FVE1	0.018	0.018	0.312	0.635	0.006	0.006	0.293	0.622	-0.014	0.007	0.031	0.120	-0.060	0.007	8.12E-16	1.08E-14	-0.052	0.007	1.94E-12	2.13E-11
FVC	0.017	0.018	0.358	0.669	0.006	0.006	0.334	0.651	0.001	0.006	0.924	0.968	-0.058	0.007	3.64E-16	5.00E-15	-0.038	0.007	7.20E-08	5.46E-07
FVE1/FVC	0.009	0.025	0.714	0.904	0.002	0.008	0.852	0.954	-0.050	0.009	3.86E-08	2.98E-07	-0.024	0.010	0.018	0.079	-0.058	0.010	1.41E-08	1.17E-07
BMI	-0.026	0.022	0.239	0.588	0.006	0.007	0.357	0.669	-8.55E-05	0.007	0.990	0.994	0.145	0.008	9.74E-77	3.06E-75	0.093	0.008	8.47E-32	1.69E-30
WHR	-0.005	0.017	0.770	0.943	-0.002	0.005	0.676	0.889	0.034	0.006	3.33E-09	2.93E-08	0.100	0.006	2.87E-55	7.88E-54	0.094	0.006	1.04E-48	2.55E-47
ApoA	0.020	0.018	0.260	0.589	0.022	0.007	0.002	0.009	0.254	0.007	3.77E-257	3.32E-255	-0.015	0.008	0.082	0.270	0.198	0.008	1.71E-121	8.34E-120
ApoB	-0.032	0.022	0.140	0.388	-0.001	0.008	0.863	0.954	0.021	0.008	0.008	0.036	0.001	0.009	0.879	0.957	0.019	0.009	0.033	0.130
TC	-0.011	0.021	0.597	0.849	0.003	0.007	0.672	0.887	0.090	0.008	9.96E-32	1.91E-30	-0.019	0.009	0.027	0.109	0.062	0.009	5.77E-13	6.51E-12
CRP	-0.021	0.019	0.281	0.612	0.005	0.007	0.438	0.730	0.013	0.007	0.074	0.254	0.065	0.008	1.33E-15	1.65E-14	0.052	0.008	2.61E-10	2.74E-09
HDL-C	0.018	0.018	0.328	0.648	0.018	0.007	0.009	0.041	0.219	0.007	5.34E-209	3.91E-207	-0.036	0.008	7.90E-06	5.19E-05	0.156	0.008	5.85E-83	2.34E-81
LPA	-0.034	0.021	0.100	0.321	0.001	0.008	0.931	0.968	-0.003	0.008	0.711	0.904	-0.004	0.009	0.696	0.895	-0.005	0.009	0.582	0.837
TG	-0.020	0.019	0.308	0.635	-0.007	0.007	0.319	0.640	-0.012	0.008	0.120	0.354	0.053	0.009	6.92E-10	6.62E-09	0.027	0.009	0.002	0.011
LDL-C	-0.016	0.022	0.449	0.738	-0.001	0.007	0.871	0.957	0.026	0.008	0.001	0.006	-0.018	0.009	0.040	0.152	0.010	0.009	0.255	0.589
Augmentation index	-0.358	0.152	0.025	0.103	-0.054	0.037	0.138	0.386	-0.028	0.043	0.519	0.797	0.030	0.044	0.493	0.777	0.019	0.048	0.692	0.893
Cardiac index	-0.091	0.190	0.635	0.867	0.041	0.041	0.311	0.635	0.082	0.048	0.084	0.276	-0.003	0.049	0.956	0.978	0.051	0.053	0.337	0.651
LV ejection fraction	-0.152	0.129	0.247	0.589	0.022	0.035	0.533	0.806	4.25E-04	0.042	0.992	0.994	-0.003	0.043	0.948	0.976	-0.009	0.046	0.838	0.953
LV end diastolic volume	0.060	0.085	0.486	0.772	-0.001	0.040	0.977	0.986	0.009	0.047	0.847	0.954	-0.051	0.048	0.290	0.622	-0.030	0.052	0.560	0.822
LV end systolic volume	0.051	0.054	0.397	0.669	-0.020	0.040	0.620	0.860	0.001	0.047	0.990	0.994	-0.059	0.048	0.222	0.568	-0.037	0.052	0.483	0.769
LV stroke volume	0.058	0.158	0.713	0.904	0.050	0.041	0.225	0.570	0.029	0.048	0.541	0.811	-0.004	0.050	0.935	0.968	0.004	0.054	0.946	0.976

***Abbreviations:** Polygenic risk score, PRS; Polygenic and gene-environment interaction risk scores, PGIRS; peak expiratory flow, PEF; forced expiratory volume in 1-second, FVE1; forced vital capacity, FVC; body mass index, BMI; waist-hip ratio, WHR; Apolipoprotein A, ApoA; Apolipoprotein B, ApoB; total cholesterol, TC; C-reaction protein, CRP; high-density lipoprotein cholesterol, HDL-C; Lipoprotein(a), LPA; triglycerides, TG; low-density lipoprotein cholesterol, LDL-C; left ventricle, LV.

Table S3. Associations of depression polygenic risk with cardiac function, lung function, and metabolic and inflammatory indicators in smoking subgroups.

	Non-smokers				Smokers							
**P=1E-5**	**PRS**				**PRS**				**PGIRS-smoke**			
**Traits**	**Beta**	**SE**	**P**	**P_{FDR}**	**Beta**	**SE**	**P**	**P_{FDR}**	**Beta**	**SE**	**P**	**P_{FDR}**
PEF	-0.007	0.004	0.103	0.325	-0.006	0.007	0.372	0.685	-0.021	0.007	0.002	0.012
FVE1	-0.006	0.004	0.136	0.385	-0.003	0.006	0.591	0.847	-0.040	0.006	5.02E-10	5.02E-09
FVC	-0.004	0.004	0.293	0.622	-0.002	0.006	0.778	0.943	-0.037	0.006	1.07E-09	1.00E-08
FVE1/FVC	-0.004	0.005	0.428	0.725	-0.004	0.008	0.616	0.860	-0.016	0.009	0.063	0.225
BMI	0.005	0.004	0.258	0.589	0.003	0.007	0.598	0.849	0.083	0.007	5.70E-34	1.25E-32
WHR	0.000	0.004	0.915	0.968	-0.003	0.005	0.557	0.822	0.054	0.006	4.45E-22	6.99E-21
ApoA	0.003	0.004	0.456	0.740	0.007	0.007	0.320	0.640	-0.025	0.007	4.08E-04	0.002
ApoB	0.003	0.005	0.772	0.943	0.005	0.007	0.501	0.785	0.001	0.008	0.869	0.957
TC	-0.002	0.005	0.664	0.887	0.011	0.007	0.109	0.338	-0.013	0.007	0.075	0.257
CRP	-0.003	0.004	0.430	0.725	0.004	0.007	0.528	0.801	0.039	0.007	3.48E-08	2.74E-07
HDL-C	-0.002	0.004	0.685	0.889	0.008	0.007	0.216	0.556	-0.035	0.007	2.88E-07	2.05E-06
LPA	-0.002	0.005	0.739	0.924	0.011	0.008	0.148	0.405	0.004	0.008	0.627	0.860
TG	0.004	0.004	0.374	0.685	0.007	0.007	0.303	0.629	0.039	0.007	1.58E-07	1.14E-06
LDL-C	-0.002	0.005	0.652	0.874	0.008	0.007	0.266	0.594	-0.009	0.008	0.228	0.574
Augmentation index	-0.025	0.026	0.336	0.651	0.030	0.038	0.424	0.724	0.077	0.038	0.046	0.171
Cardiac index	0.043	0.026	0.102	0.325	0.029	0.042	0.494	0.777	0.006	0.043	0.887	0.959
LV ejection fraction	0.002	0.024	0.928	0.968	0.044	0.037	0.235	0.581	0.009	0.037	0.819	0.948
LV end diastolic volume	0.019	0.022	0.371	0.685	-0.031	0.041	0.453	0.740	-0.027	0.042	0.527	0.801
LV end systolic volume	0.009	0.022	0.699	0.896	-0.047	0.042	0.261	0.589	-0.035	0.043	0.408	0.716
LV stroke volume	0.040	0.025	0.120	0.354	0.026	0.043	0.537	0.809	0.009	0.043	0.838	0.953
**P=0.05**												
PEF	-0.006	0.004	0.126	0.364	0.011	0.007	0.108	0.338	-0.021	0.008	0.011	0.049
FVE1	-0.004	0.004	0.273	0.606	0.008	0.006	0.173	0.462	-0.058	0.007	4.47E-15	5.32E-14
FVC	-0.002	0.004	0.684	0.889	0.006	0.006	0.298	0.625	-0.059	0.007	1.31E-16	1.87E-15
FVE1/FVC	-0.007	0.005	0.193	0.512	0.007	0.008	0.433	0.725	-0.016	0.010	0.114	0.349
BMI	-0.001	0.004	0.814	0.945	0.006	0.007	0.389	0.698	0.150	0.008	1.55E-80	5.24E-79
WHR	0.003	0.004	0.432	0.725	-0.005	0.005	0.355	0.669	0.096	0.006	2.50E-51	6.46E-50
ApoA	0.004	0.004	0.421	0.724	0.008	0.007	0.257	0.589	-0.049	0.008	1.34E-09	1.23E-08
ApoB	-0.005	0.005	0.253	0.589	-0.002	0.008	0.813	0.945	-0.001	0.009	0.883	0.958
TC	-0.005	0.005	0.314	0.637	-0.001	0.007	0.863	0.954	-0.031	0.009	2.50E-04	0.002
CRP	0.006	0.004	0.147	0.404	0.005	0.007	0.460	0.742	0.065	0.008	9.01E-16	1.17E-14
HDL-C	0.001	0.004	0.781	0.944	0.005	0.007	0.420	0.724	-0.067	0.008	1.23E-17	1.80E-16
LPA	0.000	0.005	0.935	0.968	0.003	0.008	0.721	0.909	-0.001	0.009	0.916	0.968
TG	0.001	0.004	0.801	0.945	-0.006	0.007	0.384	0.695	0.057	0.009	2.42E-11	2.59E-10
LDL-C	-0.006	0.005	0.241	0.589	-0.002	0.007	0.809	0.945	-0.022	0.009	0.014	0.061
Augmentation index	-0.073	0.026	0.005	0.026	-0.055	0.037	0.139	0.386	0.031	0.045	0.494	0.777
Cardiac index	0.040	0.027	0.130	0.374	0.031	0.041	0.449	0.738	-0.012	0.049	0.808	0.945
LV ejection fraction	-0.003	0.024	0.896	0.961	0.021	0.036	0.560	0.822	-0.006	0.043	0.893	0.961
LV end diastolic volume	0.017	0.022	0.445	0.737	-0.001	0.040	0.977	0.986	-0.047	0.049	0.330	0.649
LV end systolic volume	0.002	0.022	0.921	0.968	-0.019	0.041	0.638	0.867	-0.055	0.049	0.263	0.591
LV stroke volume	0.047	0.026	0.069	0.242	0.048	0.041	0.249	0.589	-0.004	0.050	0.930	0.968

***Abbreviations:** Polygenic risk score, PRS; Polygenic and gene-environment interaction risk scores, PGIRS; peak expiratory flow, PEF; forced expiratory volume in 1-second, FVE1; forced vital capacity, FVC; body mass index, BMI; waist-hip ratio, WHR; Apolipoprotein A, ApoA; Apolipoprotein B, ApoB; total cholesterol, TC; C-reaction protein, CRP; high-density lipoprotein cholesterol, HDL-C; Lipoprotein(a), LPA; triglycerides, TG; low-density lipoprotein cholesterol, LDL-C; left ventricle, LV.

Table S4. Associations of depression polygenic risk with cardiac function, lung function, and metabolic and inflammatory indicators in alcohol subgroups.

	Non-Drinkers				Drinkers							
**P=1E-5**	**PRS**				**PRS**				**PGIRS-alcohol**			
**Traits**	**Beta**	**SE**	**P**	**P_{FDR}**	**Beta**	**SE**	**P**	**P_{FDR}**	**Beta**	**SE**	**P**	**P_{FDR}**
PEF	0.005	0.019	0.771	0.943	-0.007	0.004	0.061	0.217	-0.005	0.004	0.252	0.589
FVE1	0.043	0.018	0.015	0.066	-0.006	0.003	0.071	0.247	-0.003	0.004	0.446	0.737
FVC	0.041	0.017	0.018	0.077	-0.004	0.003	0.209	0.542	0.006	0.004	0.109	0.338
FVE1/FVC	0.007	0.025	0.785	0.945	-0.005	0.004	0.310	0.635	-0.029	0.005	8.88E-08	6.62E-07
BMI	-0.018	0.023	0.423	0.724	0.005	0.004	0.166	0.447	0.021	0.004	7.27E-07	5.08E-06
WHR	-0.002	0.017	0.912	0.968	-0.001	0.003	0.758	0.939	0.031	0.004	1.56E-18	2.37E-17
ApoA	0.012	0.018	0.523	0.800	0.008	0.004	0.037	0.143	0.200	0.005	<0.001	<0.001
ApoB	-0.002	0.022	0.927	0.968	0.001	0.004	0.756	0.939	0.016	0.005	1.40E-03	0.008
TC	0.001	0.022	0.976	0.986	0.004	0.004	0.329	0.648	0.067	0.005	9.49E-46	2.20E-44
CRP	-0.021	0.020	0.283	0.612	-3.93E-04	0.004	0.914	0.968	-0.003	0.004	0.506	0.790
HDL-C	0.009	0.018	0.618	0.860	0.004	0.004	0.235	0.581	0.172	0.004	<0.001	<0.001
LPA	0.001	0.021	0.966	0.986	0.002	0.004	0.609	0.857	0.000	0.005	0.975	0.986
TG	0.001	0.020	0.956	0.978	0.005	0.004	0.206	0.537	-0.011	0.004	0.014	0.061
LDL-C	-0.003	0.022	0.884	0.958	0.002	0.004	0.647	0.870	0.015	0.005	1.41E-03	7.76E-03
Augmentation index	-0.276	0.147	0.070	0.246	-0.005	0.022	0.827	0.948	-0.006	0.027	0.826	0.948
Cardiac index	-0.087	0.177	0.626	0.860	0.040	0.023	0.079	0.265	0.088	0.028	0.002	0.010
LV ejection fraction	-0.033	0.123	0.791	0.945	0.013	0.020	0.514	0.796	-0.014	0.025	0.563	0.823
LV end diastolic volume	0.016	0.080	0.846	0.954	0.005	0.020	0.811	0.945	0.040	0.025	0.118	0.354
LV end systolic volume	0.031	0.051	0.550	0.820	-0.007	0.021	0.734	0.920	0.024	0.026	0.363	0.674
LV stroke volume	-0.026	0.147	0.863	0.954	0.035	0.022	0.116	0.351	0.064	0.028	0.020	0.084
**P=0.05**												
PEF	-0.023	0.018	0.194	0.512	-2.06E-04	0.004	0.955	0.978	0.001	0.005	0.855	0.954
FVE1	0.025	0.017	0.133	0.379	-0.001	0.003	0.792	0.945	0.004	0.004	0.379	0.692
FVC	0.019	0.017	0.258	0.589	0.001	0.003	0.769	0.943	0.014	0.004	0.001	0.004
FVE1/FVC	0.026	0.024	0.277	0.612	-0.005	0.004	0.296	0.623	-0.033	0.006	1.13E-08	9.52E-08
BMI	-0.026	0.022	0.225	0.570	0.002	0.004	0.593	0.847	0.022	0.005	1.42E-06	9.63E-06
WHR	-0.010	0.016	0.553	0.822	0.002	0.003	0.569	0.826	0.041	0.004	1.45E-26	2.46E-25
ApoA	0.019	0.017	0.285	0.614	0.012	0.004	0.002	0.010	0.253	0.005	<0.001	<0.001
ApoB	-0.024	0.021	0.253	0.589	-0.002	0.004	0.560	0.822	0.019	0.005	2.77E-04	0.002
TC	-0.004	0.020	0.841	0.954	-4.13E-04	0.004	0.917	0.968	0.083	0.005	1.43E-60	4.20E-59
CRP	-0.015	0.019	0.409	0.716	0.006	0.004	0.076	0.258	0.001	0.005	0.775	0.943
HDL-C	0.017	0.017	0.336	0.651	0.009	0.004	0.023	0.096	0.218	0.005	<0.001	<0.001
LPA	-0.019	0.020	0.344	0.660	0.002	0.004	0.669	0.887	-0.001	0.006	0.797	0.945
TG	-0.016	0.019	0.410	0.717	-0.001	0.004	0.806	0.945	-0.018	0.005	1.34E-04	0.001
LDL-C	-0.009	0.021	0.680	0.889	-0.003	0.004	0.460	0.742	0.018	0.005	4.99E-04	0.003
Augmentation index	-0.333	0.150	0.034	0.131	-0.059	0.021	0.006	0.030	-0.033	0.028	0.249	0.589
Cardiac index	-0.092	0.184	0.622	0.860	0.043	0.023	0.056	0.203	0.093	0.030	0.002	0.009
LV ejection fraction	-0.138	0.126	0.280	0.612	0.009	0.020	0.639	0.867	-0.023	0.026	0.387	0.698
LV end diastolic volume	0.060	0.083	0.471	0.757	0.008	0.020	0.687	0.889	0.037	0.027	0.166	0.447
LV end systolic volume	0.050	0.053	0.350	0.664	-0.009	0.021	0.679	0.889	0.024	0.027	0.390	0.698
LV stroke volume	0.059	0.153	0.702	0.898	0.049	0.022	0.028	0.113	0.056	0.029	0.055	0.201

***Abbreviations:** Polygenic risk score, PRS; Polygenic and gene-environment interaction risk scores, PGIRS; peak expiratory flow, PEF; forced expiratory volume in 1-second, FVE1; forced vital capacity, FVC; body mass index, BMI; waist-hip ratio, WHR; Apolipoprotein A, ApoA; Apolipoprotein B, ApoB; total cholesterol, TC; C-reaction protein, CRP; high-density lipoprotein cholesterol, HDL-C; Lipoprotein(a), LPA; triglycerides, TG; low-density lipoprotein cholesterol, LDL-C; left ventricle, LV.

Table S5. Significant results of mediation analysis.

Cadiac and lung function	Polygenic risk of depression	Mediator.Term	Effect	S.E.	[Boot 95% CI]	Z	P-value
**Smoking individuals**							
FVC	PGIRS-smoke	BMI.Indirect (ab)	-0.012	0.002	[-0.016, -0.009]	-7.456	8.91E-14
FVC	PGIRS-smoke	BMI.Direct (c')	-0.021	0.007	[-0.034, -0.007]	-2.993	0.00276437
FVC	PGIRS-smoke	BMI.Total (c)	-0.034	0.007	[-0.048, -0.019]	-4.539	5.65E-06
FVC	PGIRS-smoke	Waist to hip ratio.Indirect (ab)	-0.009	0.001	[-0.012, -0.006]	-6.254	4.00E-10
FVC	PGIRS-smoke	Waist to hip ratio.Direct (c')	-0.024	0.007	[-0.038, -0.011]	-3.418	0.00063037
FVC	PGIRS-smoke	Waist to hip ratio.Total (c)	-0.034	0.007	[-0.048, -0.019]	-4.539	5.65E-06
FVC	PGIRS-smoke	CRP.Indirect (ab)	-0.003	0.001	[-0.005, -0.001]	-3.416	0.00063661
FVC	PGIRS-smoke	CRP.Direct (c')	-0.030	0.007	[-0.044, -0.016]	-4.149	3.34E-05
FVC	PGIRS-smoke	CRP.Total (c)	-0.034	0.007	[-0.048, -0.019]	-4.539	5.65E-06
FVC	PGIRS-smoke	HDL-C.Indirect (ab)	-0.002	0.001	[-0.003, -0.000]	-2.477	0.01326053
FVC	PGIRS-smoke	HDL-C.Direct (c')	-0.032	0.007	[-0.046, -0.017]	-4.352	1.35E-05
FVC	PGIRS-smoke	HDL-C.Total (c)	-0.034	0.007	[-0.048, -0.019]	-4.539	5.65E-06
FVE1	PGIRS-smoke	BMI.Indirect (ab)	-0.008	0.001	[-0.011, -0.006]	-6.673	2.50E-11
FVE1	PGIRS-smoke	BMI.Direct (c')	-0.030	0.007	[-0.044, -0.016]	-4.079	4.53E-05
FVE1	PGIRS-smoke	BMI.Total (c)	-0.038	0.008	[-0.053, -0.023]	-5.095	3.48E-07
FVE1	PGIRS-smoke	Waist to hip ratio.Indirect (ab)	-0.008	0.001	[-0.010, -0.005]	-6.043	1.51E-09
FVE1	PGIRS-smoke	Waist to hip ratio.Direct (c')	-0.031	0.007	[-0.045, -0.016]	-4.154	3.26E-05
FVE1	PGIRS-smoke	Waist to hip ratio.Total (c)	-0.038	0.008	[-0.053, -0.023]	-5.095	3.48E-07
FVE1	PGIRS-smoke	CRP.Indirect (ab)	-0.003	0.001	[-0.005, -0.001]	-3.400	0.00067272
FVE1	PGIRS-smoke	CRP.Direct (c')	-0.035	0.007	[-0.050, -0.020]	-4.721	2.35E-06
FVE1	PGIRS-smoke	CRP.Total (c)	-0.038	0.008	[-0.053, -0.023]	-5.095	3.48E-07
FVE1	PGIRS-smoke	HDL-C.Indirect (ab)	-0.001	0.001	[-0.003, -0.000]	-2.402	0.01628394
FVE1	PGIRS-smoke	HDL-C.Direct (c')	-0.037	0.008	[-0.052, -0.022]	-4.941	7.78E-07
FVE1	PGIRS-smoke	HDL-C.Total (c)	-0.038	0.008	[-0.053, -0.023]	-5.095	3.48E-07
PEF	PGIRS-smoke	Waist to hip ratio.Indirect (ab)	-0.004	0.001	[-0.005, -0.002]	-4.513	6.39E-06
PEF	PGIRS-smoke	Waist to hip ratio.Direct (c')	-0.022	0.008	[-0.038, -0.006]	-2.662	0.0077575
PEF	PGIRS-smoke	Waist to hip ratio.Total (c)	-0.026	0.008	[-0.041, -0.009]	-3.101	0.00192808
PEF	PGIRS-smoke	CRP.Indirect (ab)	-0.002	0.001	[-0.004, -0.001]	-3.247	0.00116637
PEF	PGIRS-smoke	CRP.Direct (c')	-0.024	0.008	[-0.039, -0.007]	-2.835	0.00458763
PEF	PGIRS-smoke	CRP.Total (c)	-0.026	0.008	[-0.041, -0.009]	-3.101	0.00192808
**Drinking individuals**							
FVE1/FVC	PGIRS-alcohol	BMI.Indirect (ab)	0.004	0.001	[0.002, 0.006]	4.292	1.77E-05
FVE1/FVC	PGIRS-alcohol	BMI.Direct (c')	-0.032	0.007	[-0.044, -0.018]	-4.713	2.45E-06
FVE1/FVC	PGIRS-alcohol	BMI.Total (c)	-0.028	0.007	[-0.041, -0.015]	-4.083	4.44E-05
FVE1/FVC	PGIRS-alcohol	Waist to hip ratio.Indirect (ab)	0.004	0.001	[0.003, 0.005]	6.262	3.80E-10
FVE1/FVC	PGIRS-alcohol	Waist to hip ratio.Direct (c')	-0.032	0.007	[-0.044, -0.018]	-4.635	3.58E-06
FVE1/FVC	PGIRS-alcohol	Waist to hip ratio.Total (c)	-0.028	0.007	[-0.041, -0.015]	-4.083	4.44E-05
FVE1/FVC	PGIRS-alcohol	ApoA.Indirect (ab)	-0.010	0.001	[-0.012, -0.007]	-7.771	7.82E-15
FVE1/FVC	PGIRS-alcohol	ApoA.Direct (c')	-0.018	0.007	[-0.031, -0.004]	-2.617	0.0088677
FVE1/FVC	PGIRS-alcohol	ApoA.Total (c)	-0.028	0.007	[-0.041, -0.015]	-4.083	4.44E-05
FVE1/FVC	PGIRS-alcohol	TC.Indirect (ab)	0.002	0.000	[0.001, 0.002]	3.837	0.00012431
FVE1/FVC	PGIRS-alcohol	TC.Direct (c')	-0.030	0.007	[-0.042, -0.016]	-4.309	1.64E-05
FVE1/FVC	PGIRS-alcohol	TC.Total (c)	-0.028	0.007	[-0.041, -0.015]	-4.083	4.44E-05
FVE1/FVC	PGIRS-alcohol	HDL-C.Indirect (ab)	-0.010	0.001	[-0.012, -0.008]	-9.050	1.43E-19
FVE1/FVC	PGIRS-alcohol	HDL-C.Direct (c')	-0.018	0.007	[-0.031, -0.003]	-2.593	0.00951319
FVE1/FVC	PGIRS-alcohol	HDL-C.Total (c)	-0.028	0.007	[-0.041, -0.015]	-4.083	4.44E-05
Cardiac index	PGIRS-alcohol	ApoA.Indirect (ab)	0.012	0.006	[0.001, 0.024]	1.988	0.04680108
Cardiac index	PGIRS-alcohol	ApoA.Direct (c')	0.100	0.034	[0.034, 0.171]	2.897	0.00376271
Cardiac index	PGIRS-alcohol	ApoA.Total (c)	0.112	0.034	[0.042, 0.181]	3.264	0.00109699
Cardiac index	PGIRS-alcohol	HDL-C.Indirect (ab)	0.014	0.006	[0.004, 0.027]	2.494	0.01262039
Cardiac index	PGIRS-alcohol	HDL-C.Direct (c')	0.097	0.034	[0.031, 0.167]	2.835	0.00458071
Cardiac index	PGIRS-alcohol	HDL-C.Total (c)	0.112	0.034	[0.042, 0.181]	3.264	0.00109699
**Individuals who both smoke and drink alcohol**							
FVE1	PGIRS-smoke-alcohol	BMI.Indirect (ab)	-0.006	0.001	[-0.009, -0.004]	-5.397	6.78E-08

FVE1	PGIRS-smoke-alcohol	BMI.Direct (c')	-0.039	0.009	[-0.056, -0.022]	-4.383	1.17E-05
FVE1	PGIRS-smoke-alcohol	BMI.Total (c)	-0.045	0.009	[-0.063, -0.029]	-5.085	3.67E-07
FVE1	PGIRS-smoke-alcohol	Waist to hip ratio.Indirect (ab)	-0.011	0.001	[-0.014, -0.008]	-7.416	1.21E-13
FVE1	PGIRS-smoke-alcohol	Waist to hip ratio.Direct (c')	-0.034	0.009	[-0.051, -0.018]	-3.890	0.00010036
FVE1	PGIRS-smoke-alcohol	Waist to hip ratio.Total (c)	-0.045	0.009	[-0.063, -0.029]	-5.085	3.67E-07
FVE1	PGIRS-smoke-alcohol	ApoA.Indirect (ab)	0.006	0.001	[0.004, 0.009]	4.580	4.66E-06
FVE1	PGIRS-smoke-alcohol	ApoA.Direct (c')	-0.051	0.009	[-0.068, -0.035]	-5.734	9.80E-09
FVE1	PGIRS-smoke-alcohol	ApoA.Total (c)	-0.045	0.009	[-0.063, -0.029]	-5.085	3.67E-07
FVE1	PGIRS-smoke-alcohol	CRP.Indirect (ab)	-0.003	0.001	[-0.005, -0.001]	-3.004	0.00266832
FVE1	PGIRS-smoke-alcohol	CRP.Direct (c')	-0.042	0.009	[-0.059, -0.026]	-4.768	1.86E-06
FVE1	PGIRS-smoke-alcohol	CRP.Total (c)	-0.045	0.009	[-0.063, -0.029]	-5.085	3.67E-07
FVE1	PGIRS-smoke-alcohol	HDL-C.Indirect (ab)	0.008	0.001	[0.005, 0.010]	6.186	6.17E-10
FVE1	PGIRS-smoke-alcohol	HDL-C.Direct (c')	-0.053	0.009	[-0.070, -0.036]	-5.934	2.96E-09
FVE1	PGIRS-smoke-alcohol	HDL-C.Total (c)	-0.045	0.009	[-0.063, -0.029]	-5.085	3.67E-07
FVC	PGIRS-smoke-alcohol	BMI.Indirect (ab)	-0.009	0.002	[-0.013, -0.006]	-5.746	9.12E-09
FVC	PGIRS-smoke-alcohol	BMI.Direct (c')	-0.018	0.008	[-0.034, -0.003]	-2.233	0.02554201
FVC	PGIRS-smoke-alcohol	BMI.Total (c)	-0.028	0.008	[-0.043, -0.012]	-3.317	0.00090954
FVC	PGIRS-smoke-alcohol	Waist to hip ratio.Indirect (ab)	-0.013	0.002	[-0.016, -0.010]	-7.903	2.71E-15
FVC	PGIRS-smoke-alcohol	Waist to hip ratio.Direct (c')	-0.015	0.008	[-0.031, 0.000]	-1.828	0.06758459
FVC	PGIRS-smoke-alcohol	Waist to hip ratio.Total (c)	-0.028	0.008	[-0.043, -0.012]	-3.317	0.00090954
FVC	PGIRS-smoke-alcohol	ApoA.Indirect (ab)	0.009	0.001	[0.006, 0.011]	6.085	1.16E-09
FVC	PGIRS-smoke-alcohol	ApoA.Direct (c')	-0.036	0.008	[-0.052, -0.020]	-4.284	1.83E-05
FVC	PGIRS-smoke-alcohol	ApoA.Total (c)	-0.028	0.008	[-0.043, -0.012]	-3.317	0.00090954
FVC	PGIRS-smoke-alcohol	CRP.Indirect (ab)	-0.003	0.001	[-0.005, -0.001]	-3.013	0.00258364
FVC	PGIRS-smoke-alcohol	CRP.Direct (c')	-0.024	0.008	[-0.040, -0.009]	-2.961	0.00306901
FVC	PGIRS-smoke-alcohol	CRP.Total (c)	-0.028	0.008	[-0.043, -0.012]	-3.317	0.00090954
FVC	PGIRS-smoke-alcohol	HDL-C.Indirect (ab)	0.010	0.001	[0.007, 0.013]	7.588	3.25E-14
FVC	PGIRS-smoke-alcohol	HDL-C.Direct (c')	-0.038	0.008	[-0.054, -0.022]	-4.494	7.00E-06
FVC	PGIRS-smoke-alcohol	HDL-C.Total (c)	-0.028	0.008	[-0.043, -0.012]	-3.317	0.00090954
PEF	PGIRS-smoke-alcohol	Waist to hip ratio.Indirect (ab)	-0.005	0.001	[-0.007, -0.003]	-4.972	6.63E-07
PEF	PGIRS-smoke-alcohol	Waist to hip ratio.Direct (c')	-0.029	0.010	[-0.048, -0.012]	-3.020	0.00252568
PEF	PGIRS-smoke-alcohol	Waist to hip ratio.Total (c)	-0.034	0.010	[-0.053, -0.017]	-3.548	0.00038768
PEF	PGIRS-smoke-alcohol	CRP.Indirect (ab)	-0.002	0.001	[-0.004, -0.001]	-2.890	0.00385593
PEF	PGIRS-smoke-alcohol	CRP.Direct (c')	-0.032	0.010	[-0.051, -0.015]	-3.320	0.00089882
PEF	PGIRS-smoke-alcohol	CRP.Total (c)	-0.034	0.010	[-0.053, -0.017]	-3.548	0.00038768
PEF	PGIRS-smoke-alcohol	HDL-C.Indirect (ab)	0.003	0.001	[0.001, 0.005]	2.584	0.00975899
PEF	PGIRS-smoke-alcohol	HDL-C.Direct (c')	-0.037	0.010	[-0.056, -0.020]	-3.837	0.00012446
PEF	PGIRS-smoke-alcohol	HDL-C.Total (c)	-0.034	0.010	[-0.053, -0.017]	-3.548	0.00038768
FVE1/FVC	PGIRS-smoke-alcohol	BMI.Indirect (ab)	0.008	0.002	[0.005, 0.011]	5.426	5.78E-08
FVE1/FVC	PGIRS-smoke-alcohol	BMI.Direct (c')	-0.071	0.012	[-0.094, -0.047]	-5.845	5.08E-09
FVE1/FVC	PGIRS-smoke-alcohol	BMI.Total (c)	-0.063	0.012	[-0.085, -0.039]	-5.132	2.86E-07
FVE1/FVC	PGIRS-smoke-alcohol	Waist to hip ratio.Indirect (ab)	0.003	0.001	[0.001, 0.005]	2.417	0.01566121
FVE1/FVC	PGIRS-smoke-alcohol	Waist to hip ratio.Direct (c')	-0.066	0.012	[-0.088, -0.041]	-5.325	1.01E-07
FVE1/FVC	PGIRS-smoke-alcohol	Waist to hip ratio.Total (c)	-0.063	0.012	[-0.085, -0.039]	-5.132	2.86E-07
FVE1/FVC	PGIRS-smoke-alcohol	ApoA.Indirect (ab)	-0.006	0.002	[-0.010, -0.002]	-2.976	0.00291851
FVE1/FVC	PGIRS-smoke-alcohol	ApoA.Direct (c')	-0.057	0.012	[-0.080, -0.032]	-4.609	4.05E-06
FVE1/FVC	PGIRS-smoke-alcohol	ApoA.Total (c)	-0.063	0.012	[-0.085, -0.039]	-5.132	2.86E-07
FVE1/FVC	PGIRS-smoke-alcohol	TC.Indirect (ab)	0.001	0.001	[0.000, 0.002]	2.172	0.0298767
FVE1/FVC	PGIRS-smoke-alcohol	TC.Direct (c')	-0.064	0.012	[-0.086, -0.040]	-5.252	1.50E-07
FVE1/FVC	PGIRS-smoke-alcohol	TC.Total (c)	-0.063	0.012	[-0.085, -0.039]	-5.132	2.86E-07
FVE1/FVC	PGIRS-smoke-alcohol	HDL-C.Indirect (ab)	-0.006	0.002	[-0.009, -0.002]	-3.365	0.00076553
FVE1/FVC	PGIRS-smoke-alcohol	HDL-C.Direct (c')	-0.057	0.012	[-0.080, -0.032]	-4.636	3.55E-06
FVE1/FVC	PGIRS-smoke-alcohol	HDL-C.Total (c)	-0.063	0.012	[-0.085, -0.039]	-5.132	2.86E-07
FVE1	PGIRS-smoke	BMI.Indirect (ab)	-0.008	0.001	[-0.010, -0.006]	-6.582	4.65E-11
FVE1	PGIRS-smoke	BMI.Direct (c')	-0.030	0.007	[-0.046, -0.016]	-4.066	4.79E-05
FVE1	PGIRS-smoke	BMI.Total (c)	-0.038	0.008	[-0.054, -0.024]	-5.096	3.48E-07
FVE1	PGIRS-smoke	Waist to hip ratio.Indirect (ab)	-0.008	0.001	[-0.011, -0.006]	-6.344	2.24E-10
FVE1	PGIRS-smoke	Waist to hip ratio.Direct (c')	-0.030	0.007	[-0.046, -0.017]	-4.107	4.01E-05

FVE1	PGIRS-smoke	Waist to hip ratio.Total (c)	-0.038	0.008	[-0.054, -0.024]	-5.096	3.48E-07
FVE1	PGIRS-smoke	CRP.Indirect (ab)	-0.003	0.001	[-0.005, -0.001]	-3.447	0.00056605
FVE1	PGIRS-smoke	CRP.Direct (c')	-0.035	0.007	[-0.051, -0.021]	-4.731	2.23E-06
FVE1	PGIRS-smoke	CRP.Total (c)	-0.038	0.008	[-0.054, -0.024]	-5.096	3.48E-07
FVE1/FVC	PGIRS-alcohol	ApoA.Indirect (ab)	-0.007	0.002	[-0.012, -0.002]	-2.842	0.00448094
FVE1/FVC	PGIRS-alcohol	ApoA.Direct (c')	-0.046	0.012	[-0.068, -0.023]	-3.867	0.00011011
FVE1/FVC	PGIRS-alcohol	ApoA.Total (c)	-0.053	0.012	[-0.075, -0.030]	-4.563	5.03E-06
FVE1/FVC	PGIRS-alcohol	TC.Indirect (ab)	0.002	0.001	[0.000, 0.004]	2.519	0.01175591
FVE1/FVC	PGIRS-alcohol	TC.Direct (c')	-0.055	0.012	[-0.077, -0.033]	-4.752	2.02E-06
FVE1/FVC	PGIRS-alcohol	TC.Total (c)	-0.053	0.012	[-0.075, -0.030]	-4.563	5.03E-06
FVE1/FVC	PGIRS-alcohol	HDL-C.Indirect (ab)	-0.007	0.002	[-0.012, -0.003]	-3.249	0.00115677
FVE1/FVC	PGIRS-alcohol	HDL-C.Direct (c')	-0.045	0.012	[-0.068, -0.022]	-3.857	0.00011478
FVE1/FVC	PGIRS-alcohol	HDL-C.Total (c)	-0.053	0.012	[-0.075, -0.030]	-4.563	5.03E-06
FVE1	PGIRS-alcohol	Waist to hip ratio.Indirect (ab)	-0.005	0.001	[-0.008, -0.003]	-4.240	2.23E-05
FVE1	PGIRS-alcohol	Waist to hip ratio.Direct (c')	-0.015	0.008	[-0.030, 0.002]	-1.729	0.08372376
FVE1	PGIRS-alcohol	Waist to hip ratio.Total (c)	-0.020	0.008	[-0.035, -0.003]	-2.358	0.01836809
FVE1	PGIRS-alcohol	ApoA.Indirect (ab)	0.008	0.002	[0.004, 0.011]	4.392	1.12E-05
FVE1	PGIRS-alcohol	ApoA.Direct (c')	-0.028	0.009	[-0.043, -0.010]	-3.191	0.00141792
FVE1	PGIRS-alcohol	ApoA.Total (c)	-0.020	0.008	[-0.035, -0.003]	-2.358	0.01836809
FVE1	PGIRS-alcohol	HDL-C.Indirect (ab)	0.010	0.002	[0.007, 0.013]	6.294	3.09E-10
FVE1	PGIRS-alcohol	HDL-C.Direct (c')	-0.030	0.009	[-0.046, -0.013]	-3.517	0.00043645
FVE1	PGIRS-alcohol	HDL-C.Total (c)	-0.020	0.008	[-0.035, -0.003]	-2.358	0.01836809

Table S6. Five-fold Cross validation Results

Independent variable	Dependent variable	RMSE	R2	MAE	RMSE.SD	R2.SD	MAE.SD
**Smoking individuals**							
PGIRS-smoke	PEF	0.647	0.553	0.502	0.080	0.099	0.049
PGIRS-smoke	FVE1	0.094	0.990	0.062	0.024	0.004	0.017
PGIRS-smoke	FVC	0.095	0.991	0.064	0.035	0.005	0.016
PGIRS-smoke	BMI	0.713	0.330	0.510	0.320	0.165	0.106
PGIRS-smoke	WHR	0.706	0.534	0.561	0.058	0.088	0.058
PGIRS-smoke	ApoA	0.405	0.836	0.318	0.056	0.030	0.034
PGIRS-smoke	CRP	0.522	0.149	0.377	0.111	0.082	0.034
PGIRS-smoke	HDL-C	0.320	0.892	0.247	0.025	0.034	0.019
PGIRS-smoke	TG	0.594	0.628	0.453	0.149	0.105	0.059
**Drinking individuals**							
PGIRS-alcohol	FVE1/FVC	0.228	0.932	0.144	0.036	0.018	0.011
PGIRS-alcohol	BMI	0.541	0.516	0.420	0.017	0.053	0.018
PGIRS-alcohol	WHR	0.571	0.636	0.452	0.031	0.050	0.029
PGIRS-alcohol	ApoA	0.364	0.853	0.282	0.016	0.019	0.018
PGIRS-alcohol	ApoB	0.221	0.943	0.167	0.027	0.010	0.013
PGIRS-alcohol	TC	0.110	0.985	0.086	0.009	0.003	0.007
PGIRS-alcohol	HDL-C	0.241	0.933	0.192	0.020	0.007	0.018
PGIRS-alcohol	LDL-C	0.113	0.984	0.089	0.015	0.003	0.013
PGIRS-alcohol	Cardiac index	0.648	0.436	0.501	0.060	0.039	0.032
**Individuals who both smoke and drink alcohol**							
PGIRS-smoke	PEF	0.685	0.510	0.525	0.054	0.068	0.043
PGIRS-smoke	FVE1	0.098	0.991	0.064	0.023	0.003	0.006
PGIRS-smoke	FVC	0.098	0.987	0.066	0.035	0.010	0.015
PGIRS-smoke	BMI	0.627	0.366	0.478	0.159	0.197	0.082
PGIRS-smoke	WHR	0.786	0.482	0.628	0.151	0.105	0.124
PGIRS-smoke	CRP	0.539	0.157	0.391	0.101	0.166	0.042
PGIRS-smoke	HDL-C	0.290	0.909	0.229	0.036	0.019	0.038
PGIRS-smoke	TG	0.620	0.595	0.460	0.121	0.123	0.056
PGIRS-alcohol	FVE1	0.103	0.989	0.068	0.017	0.002	0.009
PGIRS-alcohol	FVE1/FVC	0.277	0.918	0.193	0.068	0.026	0.024
PGIRS-alcohol	WHR	0.730	0.538	0.570	0.122	0.109	0.094
PGIRS-alcohol	ApoA	0.438	0.813	0.349	0.053	0.050	0.042
PGIRS-alcohol	ApoB	0.265	0.922	0.202	0.044	0.032	0.025
PGIRS-alcohol	TC	0.148	0.971	0.113	0.016	0.011	0.015
PGIRS-alcohol	HDL-C	0.275	0.908	0.217	0.034	0.040	0.032
PGIRS-alcohol	LDL-C	0.143	0.977	0.109	0.015	0.006	0.017
PGIRS-smoke-alcohol	PEF	0.658	0.536	0.503	0.071	0.095	0.053
PGIRS-smoke-alcohol	FVE1	0.097	0.990	0.065	0.016	0.002	0.002
PGIRS-smoke-alcohol	FVC	0.108	0.987	0.072	0.021	0.008	0.007
PGIRS-smoke-alcohol	FVE1/FVC	0.295	0.910	0.203	0.099	0.041	0.053
PGIRS-smoke-alcohol	BMI	0.684	0.378	0.482	0.270	0.219	0.090
PGIRS-smoke-alcohol	WHR	0.709	0.543	0.559	0.052	0.068	0.051
PGIRS-smoke-alcohol	ApoA	0.414	0.821	0.328	0.056	0.053	0.049
PGIRS-smoke-alcohol	TC	0.147	0.973	0.107	0.035	0.011	0.020
PGIRS-smoke-alcohol	CRP	0.540	0.139	0.384	0.111	0.077	0.059
PGIRS-smoke-alcohol	HDL-C	0.282	0.917	0.223	0.045	0.031	0.031
